# Management and behavioral factors associated with rehoming outcomes of dogs retired from commercial breeding kennels

**DOI:** 10.1371/journal.pone.0282459

**Published:** 2023-03-02

**Authors:** Shanis Barnard, Hannah Flint, Alessia Diana, Traci Shreyer, Aitor Arrazola, James Serpell, Candace Croney

**Affiliations:** 1 Department of Comparative Pathobiology, Purdue University, West Lafayette, IN, United States of America; 2 School of Veterinary Medicine, University of Pennsylvania, Philadelphia, PA, United States of America; Universidad Santo Tomas, CHILE

## Abstract

Rehoming is a potentially stressful process for dogs retired from commercial breeding (CB) kennels, as they may struggle to cope with the myriad novel factors associated with transitioning to a home environment. Failure to adapt may increase the risk of an unsuccessful adoption, jeopardizing dog welfare and the benefits of rehoming programs. Little is known about relationships between welfare in the kennel of origin and a dog’s ability to transition to a family home. This study aimed at investigating the welfare states of dogs retiring from CB kennels in relation to varying management practices across kennels, and understanding how behavioral and management factors might be associated with rehoming outcomes. A total of 590 adult dogs from 30 US CB kennels were included in the study. Dog behavioral and physical health metrics were collected through direct observation, while management information was obtained through a questionnaire. One month after adoption, 32 dog owners completed a follow-up questionnaire (CBARQ). A principal component analysis extracted four behavioral components (PCs) which included food interest, sociability, boldness, and responsiveness. Factors such as sex, housing, breed, and the number of dogs per caretaker were reported as significant sources of variation for some of those PC scores (p<0.05). For instance, lower dog to caretaker ratio was linked to better health, sociability, and food interest scores. Significant relationships were also found between in-kennel PC scores and CBARQ scores (p<0.05). Most interestingly, higher levels of sociability in the kennel were associated with lower levels of social and non-social fear, and higher trainability after rehoming. Overall, dogs were found to be physically healthy, and a moderate proportion showed fearful responses toward either social or non-social stimuli. Results suggest that a comprehensive behavioral assessment of rehoming candidates while in the kennel may help identify dogs that may have more difficulty coping during rehoming. The implications for developing management strategies and necessary interventions that support positive dog welfare outcomes within the kennel and when rehomed are discussed.

## Introduction

The decision to retire and rehome breeding dogs is an ethical and humane choice that a growing number of US commercial breeders are electing to make. For some dogs, however, the transition from the kennel to a family home may cause distress, as they may struggle to cope with novel experiences, people and the various stimuli they may encounter en-route to their new homes as well as their novel home environment [1, 2]. Compared to dogs in shelters, which likely have some previous experience with life in a family home, breeding dogs retired from commercial kennels may face additional challenges when rehomed, as they are transferred from a consistent and predictable environment often with low or varying levels of exposure to a range of novel sensory experiences, unfamiliar people, objects, rules, surfaces, and surroundings [3]. Failure to adapt well to their new homes may increase the risk of an unsuccessful adoption, jeopardizing the dogs’ welfare as well as the benefits of well-intentioned rehoming systems. Published literature on adoption success in shelters consistently report behavioral problems as being the most likely cause of surrender or return of a dog to a shelter [4–7]. Among the most commonly reported issues are aggression toward people or other animals, social and non-social fear, house soiling, and destructiveness [4, 5, 8].

Previous findings have indicated that dogs adopted as ex-breeding stock from commercial kennels were more likely to show behavioral problems (including fearfulness, house soiling when left alone, and touch sensitivity) than those from other sources [9]. In this study, however, the conditions of the kennels of origin were unknown, and could have included unlawful or hoarding situations. In fact, the main means of recruitment for McMillan et al.’s study [9] was through animal protection and welfare organizations adopting dogs that had been confiscated from breeding facilities or those involved in ‘puppy mill rescue’. Thus, these dogs may not have been representative of those from USDA (United States Department of Agriculture)-inspected and licensed commercial breeding (CB) facilities which supply pet dogs for the U.S. Direct in-kennel assessments of lawfully operating facilities have demonstrated that a large proportion (over 70%) of dogs studied responded in an affiliative manner to an approaching unfamiliar person [10, 11]. However, half of the scored dog population responded fearfully as the tester increased the intensity of the interaction (i.e., opened the dogs’ pen doors and reached to touch them) [2, 12]. Another study found mildly fearful responses in dogs from CB kennels toward non-social stimuli (such as a plastic cone and a dog statue) [13]. How these findings collected in the kennels of origin might be associated with dogs’ behavioral responses and abilities to cope with stressors encountered during and after rehoming has yet to be explored. Studies and reports investigating the rehoming outcomes of laboratory dogs, which, like many dogs from CB kennels, often have limited exposure to novelty, found house-training and separation-related behaviors to be the main problems reported by their adopters [1, 14].

The current study provides grounds for a fundamental line of inquiry, as no evidence-based interventions or improvement can be recommended to maximize the chances that dog welfare needs are met, and of successful rehoming, until there is an understanding of current kennel management factors and how these are associated with welfare and rehoming outcomes. This is only possible through direct in-kennel assessment of dogs and follow-up after rehoming. Hence, this study achieved this by investigating the following: (1) the welfare states of breeding dogs who were candidates for rehoming; (2) new owners’ feedback on the rehomed ex-breeding dogs (1 month after adoption); (3) behavioral and management factors associated with rehoming outcomes.

## Methods

### Ethics statement

The procedures described were reviewed and approved by the Purdue University Institutional Animal Care and Use Committee (PACUC 1809001796). To protect the animals’ welfare and safety, if a dog showed extreme signs of fear or distress or behaviors that could potentially harm them or the investigators, the test was stopped. Permission to visit the CB kennels was granted by the kennel owners and a consent form for participation in the research was signed prior to the commencement of the study. Written consent to participate was also granted by the adopting owners after the dogs in the study were retired from breeding.

### Subjects and facilities

Thirty US-based (Indiana, Illinois, Ohio, Missouri and Iowa) CB kennels participated in this study on a voluntary basis. Breeders were recruited for participation through contacts within their communities, breeder meetings, and other outreach activities. Up to 20 dogs per facility that met the inclusion criteria (i.e., adults over 2 years of age, and bitches not in the last two weeks of gestation or nursing puppies) were randomly selected for testing from each facility using an online random number generator. A total of 590 dogs representing 49 breeds (including pure-breeds and designer cross-breeds) were tested (details in S1 Table in S1 File). Amongst the most represented breeds in our sample (i.e., 25 dogs or over) were Bichon Frise, Cavalier King Charles Spaniel, Golden Retriever, Labrador Retriever, Havanese, Shih Tzu and Siberian Husky.

Of the subjects, 79.7% were females (n = 470) and 20.3% were males (n = 120) (mean age = 3.62±1.47, range 2–10 years old). For each subject, information was collected about the origin of the dog (i.e., bred and raised at the kennel or purchased from another breeder as a puppy or as an adult). Often, bitches are hold-back puppies from litters bred at the kennel, while studs (breeding males) are often purchased from other kennels as puppies or as adults. Because differences in early life experiences likely shape adult dog behavior differently, we incorporated this information into our analysis. Depending on the facility, dogs were housed singly, in pairs, or in groups (3–6 dogs per pen), in pens of different sizes (all exceeding USDA requirements) and with different flooring types/materials (e.g., concrete, tenderfoot, tile), with free indoor/outdoor access and/or daily or weekly access to outdoor exercise runs. Dogs were free-fed dry kibble and had continuous access to water. Federal regulations do not currently mandate a maximum number of litters a dog may have or an age limit by which dogs should be retired from breeding. Retirement ages and adoption plans are left to the breeders’ discretion. After retirement, dogs may be rehomed directly by the breeders or through partnerships with rescue/rehoming organizations.

### Overall procedure

Dogs were assessed using a protocol that had been previously applied and validated in CB kennels by our research group [12]. The protocol was composed of a behavioral assessment (including a three-step stranger approach test and a reactivity test) and a visual physical health assessment. For feasibility purposes, the test was carried out on two consecutive days. On each day, two dogs from separate pens were closed inside their own home pen (one dog per pen) and were left for three minutes to adjust before beginning testing. On day one, one dog (randomly selected with a random number generator) would experience only the stranger approach test, while the other dog underwent the stranger-approach test immediately followed by the reactivity test. When testing for this pair ended, dogs were released, and the next pair of dogs was closed in for testing. On day two, dogs were closed in in the same order, and they would receive the opposite test, so that each dog would experience the stranger approach test twice across the two days, and the reactivity test only once. This time, before moving to the following pair of dogs, a visual health check (described below) was performed on both subjects. The whole procedure lasted 15 minutes per pair of dogs. All tests were performed by the same three trained female experimenters. The experimenter performing the test verbally communicated the scoring to a research assistant standing out of sight who recorded all scores on a portable device (i.e., Microsoft Excel spreadsheet uploaded on an iPad tablet).

At the end of each visit, breeders were administered a management questionnaire to collect information about their routine activities in the kennel and the types of interactions they had with their dogs. Finally, throughout the duration of the study (2019–2021), when tested dogs were rehomed after retiring from breeding, we sought permission from their new owners to send follow-up questionnaires about them one month after adoption.

### In kennel behavioral assessment (BA)

A detailed description of the behavioral assessment (BA) performed in the kennels can be found in Barnard et al. [12], together with validation procedures including inter-rater reliability, test-retest reliability, content validity and internal consistency. Since the dogs used in Barnard et al. [12] are a sub-sample of this study, we will not report validation metrics again, as there was good inter-rater reliability on all test items between the same three experimenters that performed the test on all the animals in this study. We also demonstrated that the day on which the BA was performed (day 1 or day 2) had no effect on the dogs’ behavioral responses to the test, and that there were no effects of the experimenter performing the test on the dogs’ responses.

The stranger approach test used was a three-step protocol developed to assess the immediate reaction of dogs to an unfamiliar person approaching and initiating an interaction with them (Table 1) [10, 12]. At each step, the dog’s reaction was recorded following the red-yellow-green (RYG) scoring system. A dog was scored ‘red’ if it showed signs of fear, aggression and/or stereotypic behavior, ‘yellow’ for ambivalent approach avoidance behavior or not clearly ‘red’ or ‘green’, and ‘green’ if it showed affiliative approach, solicited attention or was undisturbed by the presence of the experimenter. Details on all behaviors included in this scoring system can be found in Barnard et al. ([12], Table 1, p. 23)

**Table 1:** Three-step stranger approach test description and scoring system (from Barnard et al. [12]).

Step	Description	Score
1. Approach	• Experimenter approaches the pen door and scores the immediate reaction of the dog (RYG_app)	Red = 0 Yellow = 1 Green = 2
	• Tosses a treat to the dog over the kennel door and records whether or not the dog eats the treat (RYG_app/treat)	Yes = 1 No = 0
2. Open door	• Experimenter opens the pen door and scores the immediate reaction of the dog (RYG_open)	Red = 0 Yellow = 1 Green = 2
	• Offers a treat from her hand and records whether or not the dog takes the treat (RYG_open/treat)	Yes = 1 No = 0
3. Reach to touch	• Experimenter extends one hand toward the dog, while visibly holding a treat in the other hand, and scores the reaction of the dog (RYG_reach)	Red = 0 Yellow = 1 Green = 2
	• Records whether the dog allows touch (TOUCH)	Yes = 1 No = 0
	• Records whether or not the dog takes the treat from the hand (RYG_reach/treat)	Yes = 1 No = 0

In the reactivity test, the dog’s reactions to novel objects and brief social interactions were assessed. The test involved 10 steps briefly described in Table 2. The dog’s reaction was scored using a 3-point scale. In general, a score of 2 primarily reflected confident exploration/interaction with the stimuli or task; a score of 1 reflected cautious approach to/interaction with the stimuli or task; and a score of 0 reflected fearful/aggressive responses to the stimuli, or failure to engage in the task.

**Table 2:** Brief description of the steps included in the reactivity test (from Barnard et al. [12]) and duration of each step when applicable.

Step (time)	Description
1. Mat (30 sec)	Reaction to a rubber mat placed on the pen floor with a treat on top. The mat was left in the pen for the remainder of the test.
2. Leash (30 sec)	Reaction to a slip leash placed on one side of the mat with a treat on top. The leash was left there for the entire test to familiarize the dog with it before looping it over his/her head (subtest 10).
3. Cone (30 sec)	Reaction to a plastic traffic cone placed on the mat. The cone and all subsequent objects were removed after scoring.
4. Problem solving (30 sec)	Success in retrieving (or not) a treat placed on the mat and under an upside-down bowl.
5. Squeaky toy	Initial and final reaction to the sound of a plastic squeaky dog toy squeezed in front of the dog for up to 10 times.
6. Ball toy (30 sec)	Reaction to a rubber ball placed on the mat.
7. Artificial dog (30 sec)	Reaction to a realistic dog statue placed on the mat.
8. Umbrella	Initial and final reaction to an umbrella opened in front of the dog for up to 10 times.
9. Response to cues	Reaction to the experimenter first calling the dog (‘*come’*) and then luring him/her to sit using a treat (‘*sit*’). Each cue was repeated a maximum of two times; the treat was offered at the end of the cues regardless of the dog response.
10. Loop leash	Reaction to the experimenter showing the leash in an open loop and attempting to slip the leash over the dog’s head. A treat was used to lure the dog inside the loop. The dog could retreat at any time as the loop was never closed around the dog’s neck.

### In-kennel physical health assessment

On the last day of the visit, upon completing the behavioral assessment, the experimenter assessed by visual examination (i.e., the experimenter standing outside the dog’s pen with no handling of the animal) the physical health of each dog. This assessment was based on the previously validated FIDO tool metrics [2, 15]. The metrics scored included body condition score (BCS) on a 1–5 scale (e.g., 1 = severely underweight, 3 = normal, 5 = obese), body cleanliness (i.e., percentage of body covered in debris, 1 = 0% no debris; 2 = less than 25% debris on all four paws maximum; 3 = 26–50% debris on legs, chest and abdomen; 4 = 51–75% debris on legs, chest, abdomen and topline; 5 = >76% debris on legs, chest, abdomen, topline, neck, and head), tear staining (0 = absent, 1 = mild, 2 = moderate, 3 = severe) and finally presence/absence (1–0) of nasal or ocular discharge, sneezing, coughing, poor coat condition, wounds/lesions or lameness.

### Management questionnaire

The questionnaire was administered by an experimenter who read the questions to the breeders and recorded their answers on a printed copy of the survey. The questionnaire was designed to minimize social desirability bias. For example, the same questions were asked in different ways, and questions were formulated so as to not to lead the respondents’ answers. Where feasible, scaled responses permitted answers that were accurate without being so specific as to elicit non-responses, or those that might otherwise have been viewed as desirable to the experimenter. In addition, respondents were repeatedly reminded of the confidential nature of the survey and that there were no correct or incorrect responses. Finally, the survey was administered at the end of the second day of data collection to allow the breeder to feel more comfortable interacting candidly with the experimenters. In the first section of the survey, breeders were asked if they performed specific management practices (including exercise, environment enrichment, training, puppy socialization, and adult socialization) never/rarely (i.e., not at all/too infrequently to accurately document); sometimes (i.e., every other week or a few times per month); often (i.e., at least two days per week); or always (i.e., daily). Information on the kennel size (i.e., total number of adult dogs in the facility) and housing type (i.e., if dogs were housed individually, in pairs or group) was also collected. The second section of the survey was used to record detailed information about the specific types of care and management practices used with or provided to the dogs, such as types of enrichment or exercise, and how the dogs were handled and managed. Most of these questions were binary (i.e., task/action performed yes/no) with a follow up question on frequency: daily (5–7 days per week), weekly (1–2 times per week), occasionally (few times per month or sporadically), and space for comments if needed. A complete copy of the survey is provided in the S2 File. These questions were ultimately categorized to create the following management factors used for analysis: environmental enrichment, socialization with adult dogs, socialization with puppies, quality of handling (i.e., use of gentle/low-stress handling techniques [16]), exercise, and training. Finally, a dog to caretaker ratio for husbandry (dog:husbandry)—i.e., the total number of dogs in the kennel divided by the number of caretakers reported to provide routine husbandry was calculated. Similarly, a dog to caretaker ratio for positive interactions (dog:interaction)—i.e., the total number of dogs in the kennel divided by the number of caretakers reported to provide other types of social interaction, including play and training was calculated. Children within the household were also included in this last count as they were often involved in these activities.

### Rehoming questionnaire

One month after adoption, the new owners were asked to complete a survey on the physical and behavioral conditions of the dog. The survey was composed of two parts. The first part inquired about whether the dog had any health or behavioral problems. Owners were also asked if they were satisfied with the dog and if the dog met their expectations. To better understand how the dogs were behaving in their new homes and if any behavioral problems were occurring, in the second part of the survey, the Canine Behavioral Assessment and Research Questionnaire (CBARQ) was used [17, 18]. Briefly, the CBARQ queries the dog’s behavior in response to common daily events or situations relating to the following 15 behavioral sub-scales: stranger-directed aggression, stranger-directed fear, owner-directed aggression, dog-directed aggression, dog-directed fear, dog rivalry (i.e., aggression toward familiar dogs), non-social fear, trainability, separation-related problems, touch sensitivity, attachment/attention-seeking, excitement, energy, chasing and miscellaneous. A description of the single questionnaire items included in each factor can be found in Duffy et al. (p. 102–103) [18].

### Statistical analysis

All statistical tests were carried out using IBM SPSS (v.24) software and SAS 9.4 (SAS Institute Inc., Cary, NC, USA) with an alpha < 0.05 used to indicate significant differences.

#### In-kennel behavioral assessment

Descriptive statistics on dogs’ general behavioral reactions to different components of the behavioral assessment were reported. The behavioral variables from the BA were then grouped and reduced by using a principal component analysis (PCA) with varimax rotation, and components with Eigenvalue >1 were extracted. Kaiser-Meyer-Olkin (KMO) and Bartlett test of Sphericity were checked to ensure data assumptions were met. Principal components (PC) scores (i.e., standardized scores calculated using the least squares regression procedure in SPSS) were created for each extracted behavior component to allow further analysis.

#### In-kennel physical health assessment

Individual health metrics collected in the kennels were assessed descriptively and a total health score was created by transforming categorical variables into binary scores (BCS normal = 0, underweight or overweight = 1; cleanliness score 0% = 0, any presence of debris = 1; tear staining none = 0, presence = 1) and adding all the health scores together.

#### Management questionnaire

The first section of the management survey was used to provide a descriptive overview of the management practices in place at the sampled kennels. Each item from the second section of the management questionnaire was converted to a binary score (1–0) or an ordinal scale in case of frequency (e.g., never = 0, occasionally = 1, weekly = 2, daily = 3) where higher scores represented more desirable practices. They were then added by category to form a final factor score for the practices: environment enrichment, socialization of adult dogs, socialization of puppies, quality of handling, exercise, and training. These management factors and the dog to caretaker ratios were ultimately used for statistical analysis.

#### Sources of behavioral and health variation within kennels

To explore the sources of variation in the dogs’ behavioral and health metrics, cross-classified multilevel models in MIXED procedure of SAS were used. Model assumption of residuals normality was met by checking skewness and kurtosis and visual examination of normality plots. Breeds with less than five dogs were removed from the dataset yielding a total of 550 dogs from 31 breeds. Age of the dogs was re-coded into three categories: young (i.e., <4 years old), medium (i.e., 4>age<5 years old) and old (i.e., >5 years old). Behavioral PC scores and the total health scores were used as dependent variables. Age, sex, origin (i.e., whether the dog was raised at the kennel or purchased as a puppy or as an adult) and housing type (i.e., if dogs were housed individually, in pairs or group) were included as categorical fixed effects, and the variables dog:husbandry and dog:interaction were included in the model as covariates. Facility and breed were included as random effects. Results of categorical fixed effects are presented as least squares means ± SE, those for the covariates are presented as regression coefficient ± SE while results of random effects are presented as estimates ± SE. A Tukey–Kramer adjustment was used to account for multiple comparisons. The criterion for statistical significance was established at p<0.05 and statistical trend was set at p>0.05 and p<0.10.

#### Rehoming questionnaire

Descriptive statistics of the new owners’ satisfaction scores and overall reported health of the dogs at 1 month after adoption were reported. Following Duffy et al. [18], the CBARQ subscale scores (all but ‘miscellaneous’) mentioned above were calculated by averaging the scores of the representative questionnaire items for each subscale. When an item was not recorded by the owner it was considered a missing value, and the subscale score was averaged for the remaining items (up to 25% of missing items per subscale allowed). Final subscales with missing values of over 30% were explored descriptively but excluded from statistical analysis. Within the ‘miscellaneous’ subscale, we analyzed descriptively the items related to ‘house soiling’ and ‘escape’, being of greater interest for this study.

#### Behavioral and management factors associated with rehoming outcomes

Multiple regression models in GLM procedure of SAS were performed to explore the association between behavioral assessment in the kennel (i.e., PC scores) and CBARQ outcomes. Model assumptions of residuals normality was met, by checking skewness and kurtosis and visual examination of the normal plot. Results are presented as regression coefficient ± SE. The criterion for statistical significance was established at p<0.05 and statistical trend was set at 0.05>p<0.10. An exploratory analysis revealed no effect of management on outcome measures using continuous scales, so we used percentiles to select the facilities in the lower 25th percentile and upper 75th percentile scores for each management category. We used non-parametric Mann-Whitney U test for independent groups to evaluate if selected CBARQ scales differed between high and low standard practices.

## Results

### In-kennel behavioral assessment

**Fig 1:**
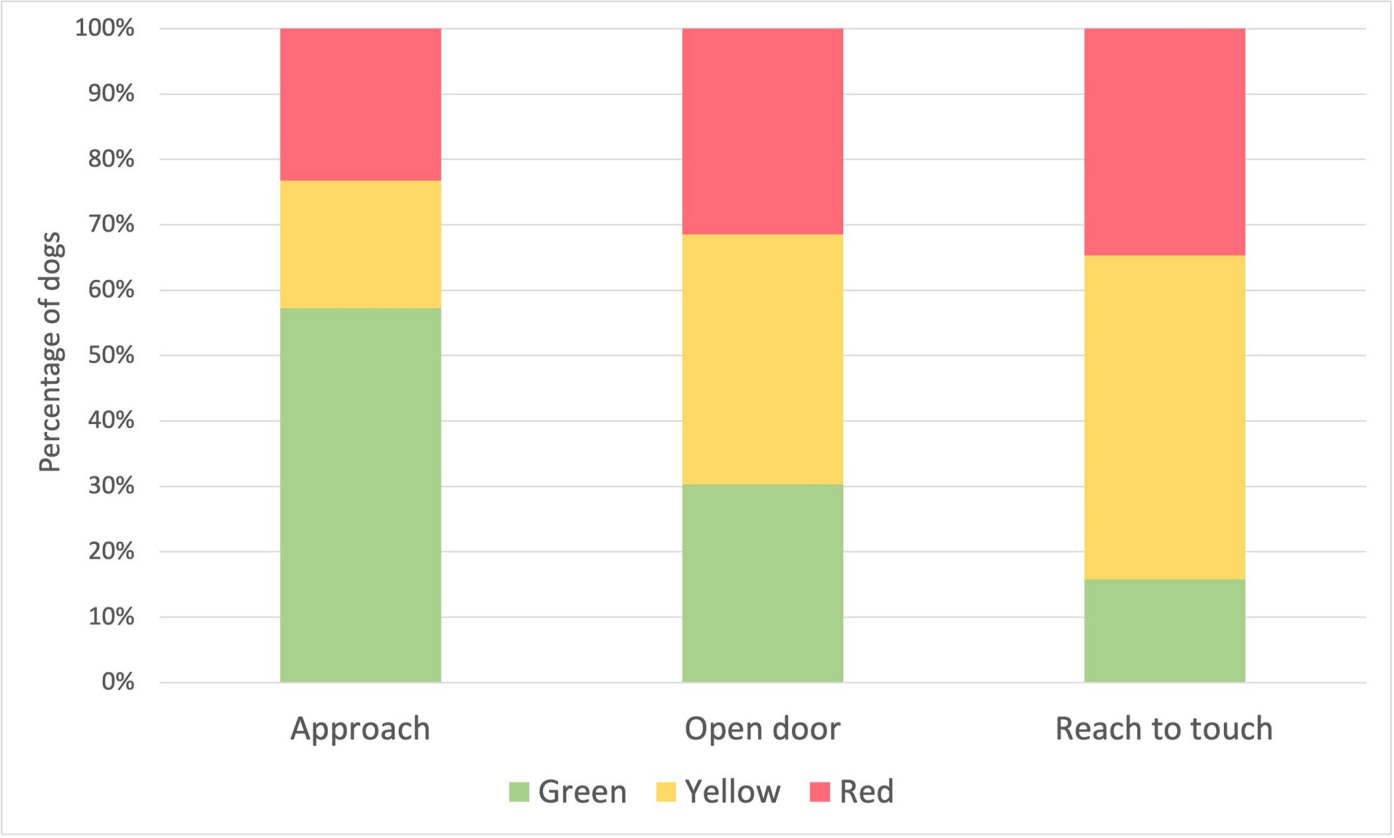
Dogs’ responses to the approach test. Percentage of tested dogs (total n = 589) scoring ‘red’ (showed signs of fear, aggression and/or stereotypic behavior), ‘green’ (showed affiliative approach, solicited attention or was undisturbed by the presence of the experimenter) or ‘yellow’ (showed ambivalent approach-avoidance behavior, or were not clearly ‘red’ or ‘green’) during the three-step approach test (i.e., approach, open door and reach to touch).

In response to a stranger approaching them from outside of their pens, more than half of the tested dogs showed non-fearful and/or affiliative behavior (57.3% ‘green’). As predicted, dogs showed increasingly fearful responses (especially ambivalent ‘yellow’ reactions, from 19.5% to 49.5%) as the interactions became more intense (i.e., the pen door was opened and the stranger reached to touch the dog, Fig 1). A fearful response (‘red’) was observed for 24–34% of dogs across all three steps of the test. Very few dogs (n = 16, 2.7%) showed any signs of aggression (i.e., growling, rigid/tense body posture) during this test. When reaching inside the pen, 46.3% of dogs allowed the tester to touch them. In total, 30 dogs (5.1%) showed signs of repetitive behavior including circling, pacing, or wall-bouncing.

**Fig 2:**
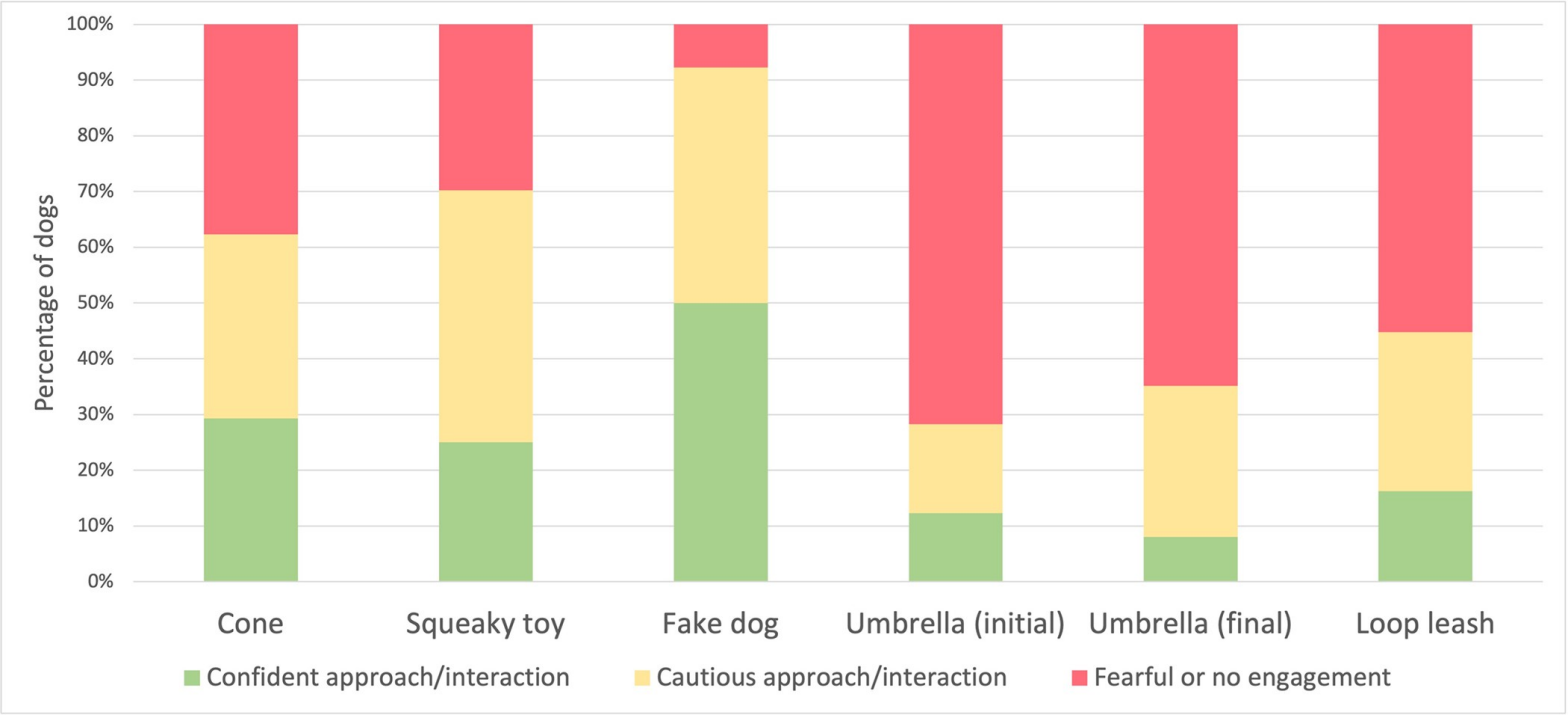
Dogs’ responses to non-social stimuli. Percentage of tested dogs (total n = 584) approaching or interacting with the stimulus in a confident manner (green), approaching or interacting with the stimulus in a cautious manner (yellow) or not engaging with the stimulus or showing a fearful response (red) when exposed to different stimuli such as a plastic traffic cone, a squeaky dog toy, a fake dog, an opening umbrella, or a loop leash placed around the neck.

During the behavioral assessment, dogs were also exposed to a range of novel objects. Fig 2 illustrates how some stimuli elicited more fearful responses (e.g., opening of an umbrella) than others (e.g., presentation of a fake dog).

A PCA was used to reduce the number of behavioral variables generated by the behavioral assessment which extracted four main principal components (PCs) with Eigenvalue >1, explaining 69% of the total variance (Table 3). KMO = 0.95, and a Bartlett test of Sphericity was significant (p<0.0001).

**Table 3:** Principal components extracted by the principal component analysis (PCA). Loadings higher than 0.50 are in bold.

Variables	Component			
	PC1_food interest	PC2_sociability	PC3_boldness	PC4_responsiveness
Response to cues (*sit*)/treat	**.791**	.340	.135	.245
Response to cues (*come*)/treat	**.757**	.391	.181	.192
Loop leash/treat	**.703**	.271	.103	.417
Response to cues (*sit*)	**.689**	.474	.199	.237
Leash/treat	**.668**	.009	.477	.144
Mat/treat	**.663**	.105	.460	.080
RYG_reach/treat	**.633**	**.586**	.104	.021
RYG_open/treat	**.625**	**.563**	.126	.021
RYG_app/treat	**.589**	.315	.274	-.114
Problem solving	.464	.106	.432	.174
TOUCH	.265	**.763**	.122	.170
RYG_open	.215	**.753**	.315	.236
RYG_reach	.249	**.749**	.243	.260
RYG_app	.201	**.639**	.429	.100
Response to cues (*come*)	.361	**.594**	.370	.349
Squeaky toy (initial)	.293	**.580**	.331	.302
Squeaky toy (final)	.312	**.572**	.322	.301
Loop leash	.286	**.532**	.259	**.511**
Leash	.307	.259	**.767**	.182
Mat	.193	.226	**.763**	.144
Artificial dog	.063	.209	**.685**	.273
Ball toy	.281	.297	**.601**	-.033
Cone	.189	.372	**.577**	.355
Umbrella (initial)	.113	.227	.149	**.807**
Umbrella (final)	.143	.214	.246	**.802**
Explained Variance (%)	21.2	21.0	16.1	10.7

The first component encompassed all items involving treats, and thus, was labelled PC1_food interest. The second component was labelled PC2_sociability as scores related to the dogs’ responses to closer interactions with the experimenter loaded here (e.g., responses to the approach test, ‘come’ cue, interaction with a squeaky toy handled by the tester, and the response to looping the leash over the dog’s head). Accepting a treat during the open and reach steps of the approach test also cross-loaded on this factor. The third component was comprised of scores related to the dogs’ responses to inanimate stimuli (such as the cone, the artificial dog, the leash, the mat, and the ball toy) and was labelled PC3_boldness. The loading for the problem-solving test was not high enough to make the cut-off of 0.50. Nevertheless, it loaded higher on this as well as the first component (PC1_food interest). The fourth and last component included responses to the opening of the umbrella, so it was labelled PC4_responsiveness. The leash loop test also cross-loaded here.

### In-kennel physical health assessment

**Fig 3:**
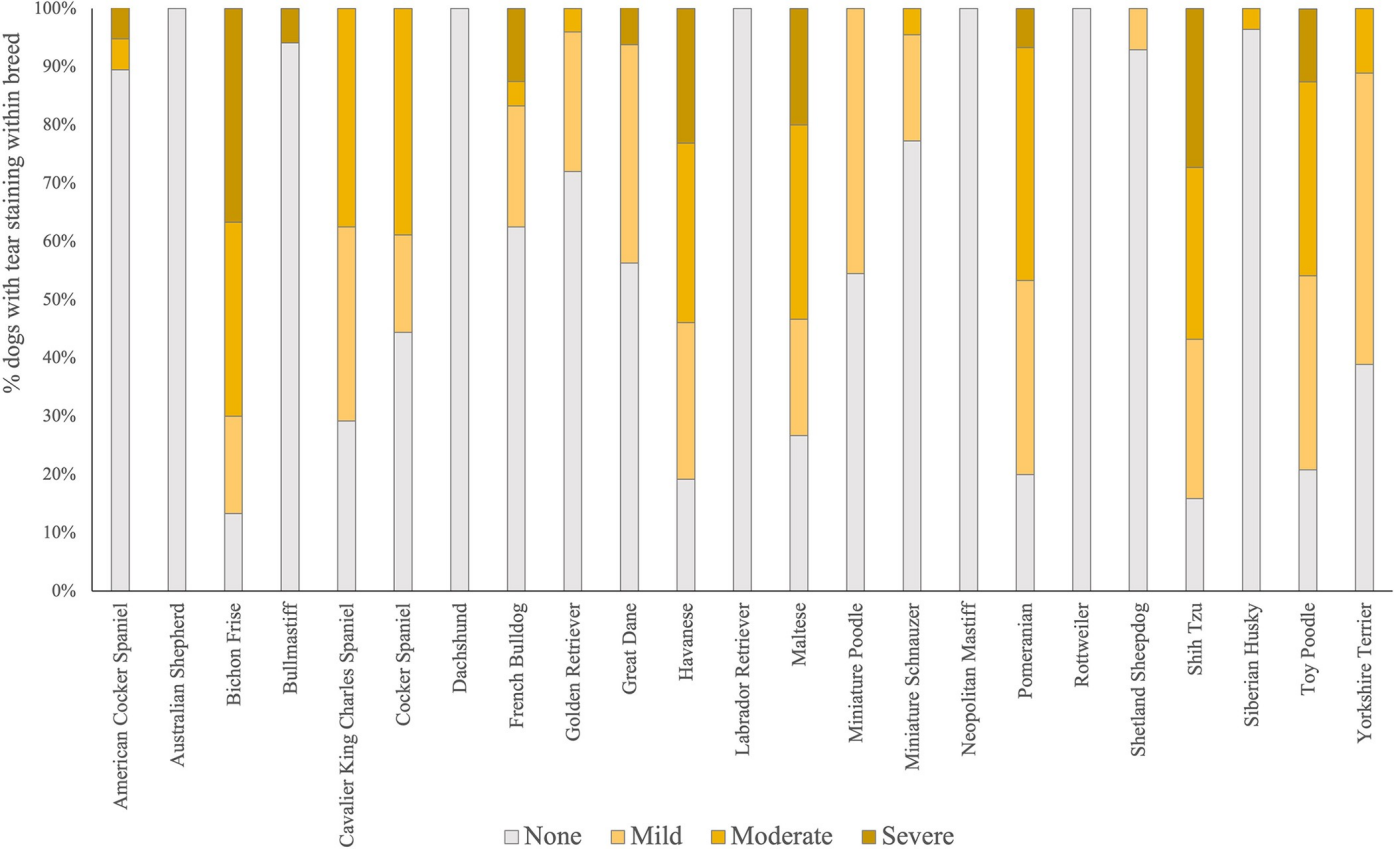
Tear staining severity by breed. Percentage of dogs (within breed) showing different levels of tear staining (i.e., none, mild, moderate or severe). Note: only breeds in our sampled population with more than 10 individuals are presented here (n = 502), see S1 Table in S1 File for reference of sampled breeds.

Physical health measurements highlighted that the dogs in our sample were generally healthy. Ocular discharge was reported on 18.2% of dogs. No sneezing or coughing was reported. Fewer than 3% of dogs had observable wounds, poor coat condition, lameness, or nasal discharge. A portion of the dogs (23.8%) were scored as having ‘other’ health problems which for the most part included observed discoloring of the coat, especially the paws, which is often attributed to excessive licking of the paws (although this behavior was never observed). Most dogs were, clean (80.1%), 17% had debris on less than 25% of their bodies, and 2.9% had debris on a larger proportion of their body. One of the most frequently recorded item was tear staining. Forty one percent of dogs had some level of tear staining, but this was mostly breed dependent (Fig 3).

Finally, 72.5% of dogs had optimal BCS (score 3), 24.6% were overweight (score 4 or 5) and only 2.9% (n = 17) were scored as underweight (score 2). No dogs were severely underweight (score 1). When calculating the total health score by adding the total frequency of observed issues for each dog, 23.3% of animals had no recorded health concerns, 24.6% had only one, and the remaining dogs had two or more recorded concerns (mean = 1.88, median = 2).

### Management questionnaire

Management information was analyzed for 29 kennels. We were not able to collect management information from one breeder. Half of the kennels enrolled (52%) were of medium size (40 to 80 adult dogs), 27% of them were small (fewer than 40 adult dogs) and 21% were larger kennels with over 80 adult dogs. The type of housing differed between kennels (often associated with the breed being small or large) and often males and females were housed differently, with females mostly housed in groups (18/29 kennels) and males housed in groups (14/29) or singly (9/29; S1 Fig in S1 File).

**Fig 4:**
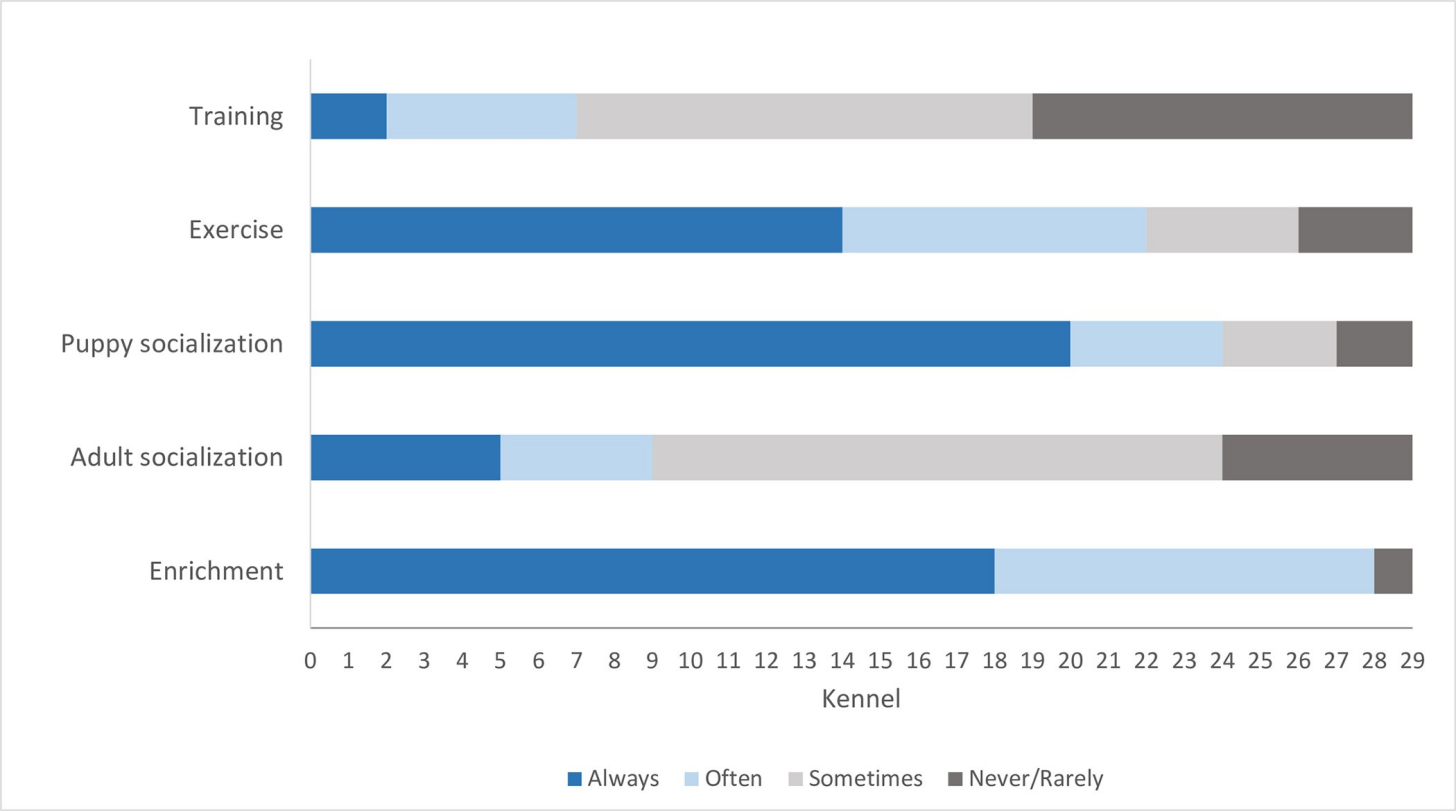
Management practices. Number and frequency of kennels performing specific behavioral management practices.

On average, the dog to caretaker ratio for husbandry (dog:husbandry) was 23.7 dogs/caretaker, SD = 14.6, range 7–75; whereas the average dog to caretaker ratio for positive interaction (dog:interaction) was 26.3 dogs/caretaker (this includes adolescents and children), SD = 21.6, range 3.5–85. Twenty-four breeders reported that their dogs had regular exposure to children under 12 years of age which would play and interact with the dogs regularly. Fig 4 illustrates the responses of breeders to the general questions of the first section of the survey.

Practices such as providing exercise, enrichment and puppy socialization were found to be used regularly by most breeders. For example, in 86.2% of the facilities, dogs had unrestricted indoor/outdoor access in their home pens. In 55.2% and 20.7% of facilities, dogs had additional access to outdoor exercise yards daily or weekly, respectively. All facilities provided chews to the dogs as enrichment and 86.2% also provided different types of dog toys. Gentle handling of the puppies in the first three weeks of life occurred daily at 58.6% of facilities, and another 34.5% provided early handling a few times per week.

Training and target adult socialization were more sporadic. For example, 58% of breeders reported that they leash trained dogs occasionally (normally only some dogs and only before they were rehomed) as dogs are normally moved between areas by carrying them or by corridor systems that lead them to the desired areas. Further, 31% of breeders occasionally performed basic manner training to the dogs. In 89.7% of facilities, dogs reportedly saw unfamiliar people in the kennel only occasionally. Only 34.5% of breeders positively interacted with the dogs daily outside the normal feeding-cleaning routine, while 24.1% and 41.4% reported weekly or only occasional additional interactions with the dogs, respectively.

### Sources of behavioral and health variation within kennels

Sex had a significant effect on PC1_food interest (p < 0.0001), with females being likely to score higher (i.e., more likely to eat food) on this PC score than males (females = 0.096 ± 0.094, males = -0.348 ± 0.118). The dog:interaction ratio also affected the PC1 score (Table 4). A lower PC1 score was found when the number of dogs per caretaker was higher (p = 0.015). Housing and the dog:husbandry ratio had a significant effect on PC2_sociability (Table 4), with dogs housed individually scoring higher (i.e., more likely to show affiliative behavior) than the other dogs (p = 0.033). Also, a lower PC2 score was found when the number of dogs per caretaker providing routine husbandry was higher (p = 0.003). Origin of the dog had a significant effect on PC3_boldness (p = 0.011), with dogs purchased as puppies scoring higher (i.e., more likely to explore and less fearful to interact with stimuli) on this component than dogs purchased as adults and dogs bred and raised at the kennel. Sex and housing had a significant effect on PC4_responsiveness (Table 4). Male dogs were somewhat more likely to score higher on responsiveness (i.e., more confident response towards a startling stimuli) than females (males = 0.322 ± 0.107, females = 0.092 ± 0.073; p = 0.046). Dogs housed singly or in pairs scored higher than group-housed dogs (p = 0.004). Finally, the dog:interaction ratio had an effect on the total health score (Table 4), where a higher health score (i.e., more health issues) was associated with a higher number of dogs per caretaker (p = 0.001). Age had no significant effect on any PC score.

**Table 4:** Least squares means (LS mean) and SE of the effect of sex, origin, age, housing, dog:husbandry ratio and dog:interaction ratio on PC scores and total health.

		PC1_food motiv.		PC2_sociability		PC3_boldness		PC4_responsiveness		Tot health	
Variable		LS mean	SE	LS mean	SE	LS mean	SE	LS mean	SE	LS mean	SE
**Sex**											
	Female	0.096^a^	0.094	0.007	0.100	0.089	0.094	0.092^a^	0.073	1.416	0.129
	Male	-0.348^b^	0.118	0.081	0.121	0.052	0.118	0.322^b^	0.107	1.329	0.149
**Origin**											
** **	Raised	-0.075	0.119	-0.029	0.122	-0.056^a^	0.118	0.111	0.102	1.309	0.150
** **	Purchased (puppy)	-0.185	0.119	0.033	0.121	0.277^b^	0.120	0.165	0.106	1.380	0.149
** **	Purchased (adult)	-0.117	0.105	0.128	0.109	-0.009^a^	0.104	0.345	0.088	1.429	0.137
**Age** (years)											
** **	2–3	-0.011	0.097	0.150	0.103	-0.020	0.095	0.079	0.077	1.231	0.131
** **	4–5	-0.194	0.107	-0.032	0.111	0.069	0.106	0.225	0.091	1.316	0.138
** **	>5	-0.172	0.148	0.014	0.149	0.164	0.149	0.316	0.139	1.572	0.177
**Housing**											
(N dogs in pen)	1	-0.126	0.153	0.262^a^	0.155	0.088	0.155	0.308^a^	0.129	1.404	0.188
** **	2	0.007	0.119	-0.152^b^	0.121	0.073	0.116	0.322^a^	0.098	1.389	0.150
** **	3 or more	-0.257	0.109	0.022^a,b^	0.114	0.051	0.108	-0.009^b^	0.087	1.325	0.143
**dog:husbandry** ^**1**^		NI		-0.0153±0.0051*		NI		NI		NI	
**dog:interaction** ^**1**^		-0.0086±0.0035*		NI		NI		0.0036±0.0024		0.0133±0.0040*	

^a,b^ Significant difference within traits of predictor variables; P < 0.05

^1^Results for continuous covariates presented as regression coefficient ± SE; ^⁎^P < 0.05

NI = Not included in the final model

There were significant differences among facilities for PC1 (0.108 ± 0.046; p = 0.009), PC2 (0.087 ± 0.048; p = 0.034), PC3 (0.132 ± 0.051; p = 0.005) and total health (0.128 ± 0.055; p = 0.010), indicating a substantive variability of the behavioral and health scores among CB kennels (S2 Table in S1 File). Significant differences were also observed among breeds (S3 Table in S1 File) but only for PC2 (0.090 ± 0.047; p = 0.027) and total health (0.211 ± 0.082; p = 0.005). Breeds with health scores significantly higher (i.e., higher reported prevalence of health issues) compared to the intercept were the Bichon Frise, Cavalier King Charles Spaniel, Cocker Spaniel, Shih Tzu and Toy Poodle. At the opposite end, breeds with lower health scores (i.e., low to no prevalence of health issues) were the Dachshund, and the Shiba Inu. PC3 showed a tendency to differ between breeds (0.041± 0.027; p = 0.065). A graphic representation of the between and within breed variation on PC2_sociability for breeds that were represented by 10 or more individuals in our sample is presented in the S2 Fig in S1 File.

### Owners’ feedback on rehomed dogs at 1 month after adoption

We were able to collect follow up information in the form of owner reports for 32 of the dogs tested at the kennels (1 month after rehoming, mean age 4.5 years old, range 2–8 years old). None of the rehomed dogs included in the sample were returned after adoption during the study timeframe. This sample included 4 males (12.5%) and 28 females (87.5%). A range of breeds were represented as follows: Cavalier King Charles and Cocker Spaniel (n = 5 individuals per breed); Golden Retriever (n = 4); Maltese and Yorkshire Terriers (n = 3 per breed); French Bulldog, Pomeranian and Toy Poodle (n = 2 per breed); Bichon Frise, Great Dane, Labrador Retriever, Lhasa Apso, Miniature Schnauzer and St. Bernard (n = 1 per breed). All dogs were neutered/spayed before they were placed to a new home.

At one month after adoption, overall satisfaction level was very high: 87.1% were very satisfied and 12.9% were satisfied. No owner scored 3 (neutral) or below satisfaction. Most owners rated the dog as far exceeding (38.7%) or exceeding (35.5%) expectation; 19.4% reported the dog meeting their expectations (some of these owners already had experience with retired breeding dogs); 6.5% declared the dog below expectation. Interestingly, even when a dog was rated below expectation, the satisfaction score was still very high.

#### Physical health of rehomed dogs

**Fig 5:**
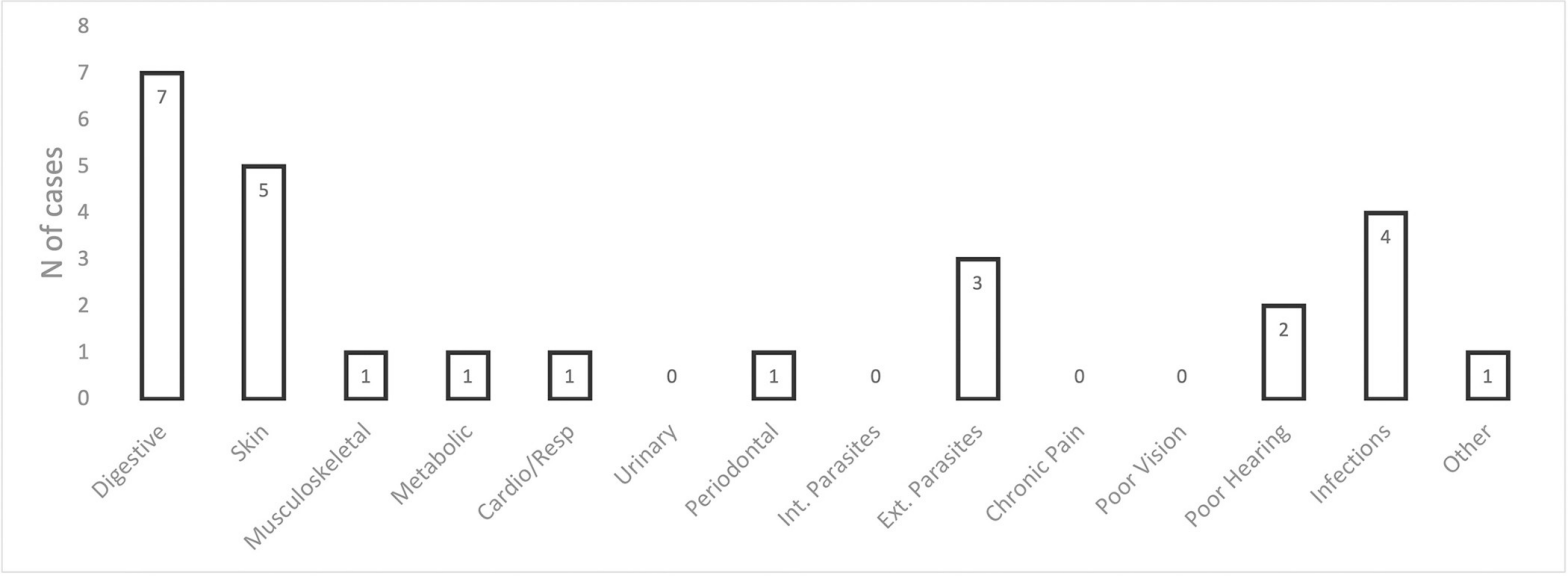
Physical health of rehomed dogs. Number of cases of health concerns reported by owners during the first month after adoption (n = 32).

One month after rehoming, we asked the new owners to report any health problems their dogs experienced after joining their family. More than half of the rehomed dogs (53.1%) reportedly showed no evidence of health problems. For the remaining dogs, Fig 5 shows the types of concerns reported, with diarrhea (digestive), skin problems and infections (especially ear infections) being the most common.

#### Behavioral assessment of rehomed dogs

**Fig 6:**
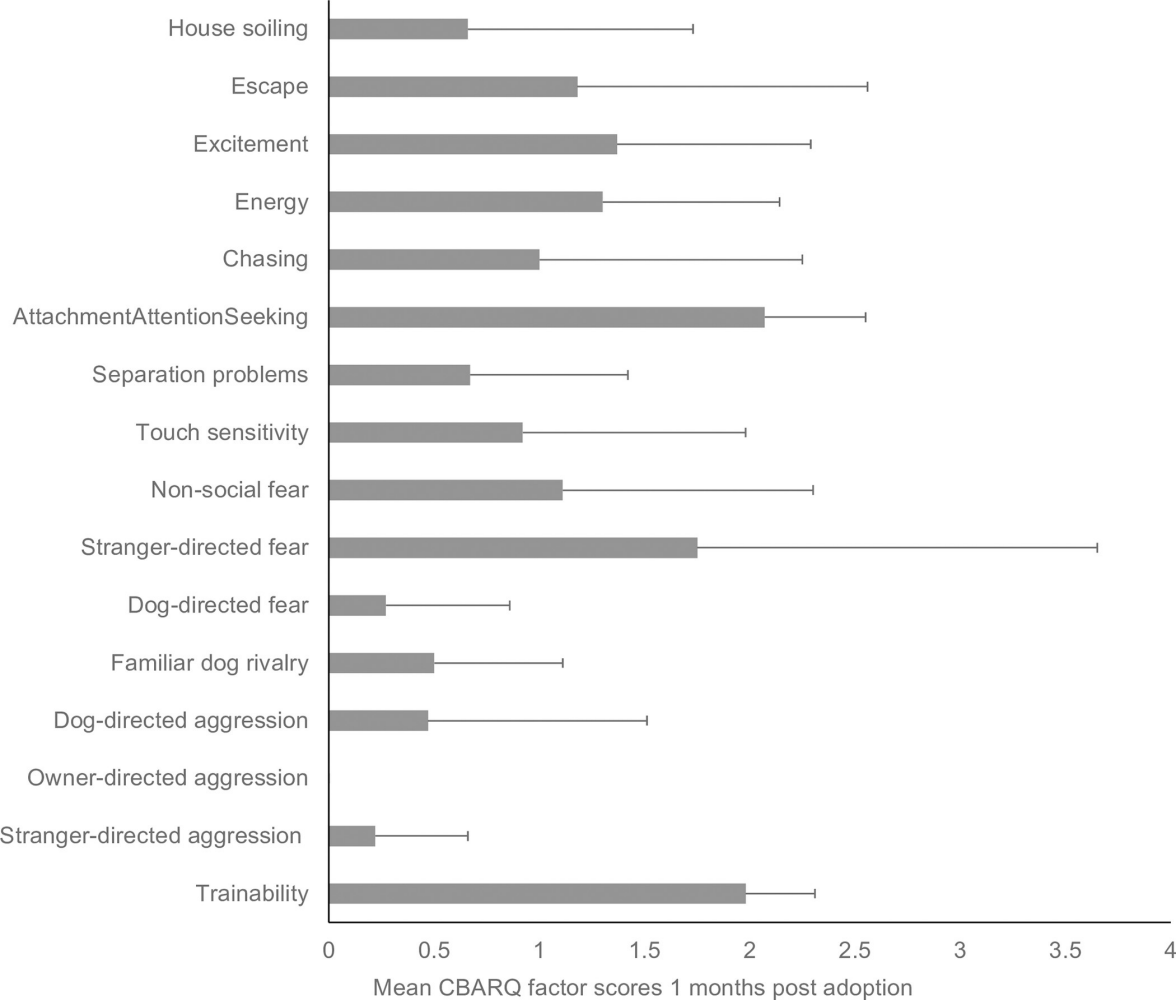
Mean CBARQ scores for selected subscales. Mean statistics of the CBARQ behavioral sub-scale scores, and of relevant CBARQ items included in miscellaneous (i.e., house soiling, escape) obtained from the owners of the 32 rehomed dogs 1 month after adoption. CBARQ scale ranges from 0–4 where 0 is a behavior not observed and 4 is a behavior often observed in the dog.

Results from owner reports allowed us to estimate the main reported behavioral problems in this population of retired breeding dogs. As illustrated in Fig 6, at one month after adoption, on a range from 0–4 the CBARQ behavioral subscale attachment/attention-seeking had the highest mean score (mean±SD = 2.07 ± 0.48), followed by trainability (1.98 ± 0.33) and stranger-directed fear (1.75 ± 1.90). Conversely, there was no reported owner-directed aggression (mean = 0) or stranger-directed aggression (0.22 ± 0.44). Dog-directed fear (0.27 ± 0.59) and dog-directed aggression (0.47 ± 1.04) were rarely reported.

### Behavioral and management factors associated with rehoming outcomes

**Fig 7:**
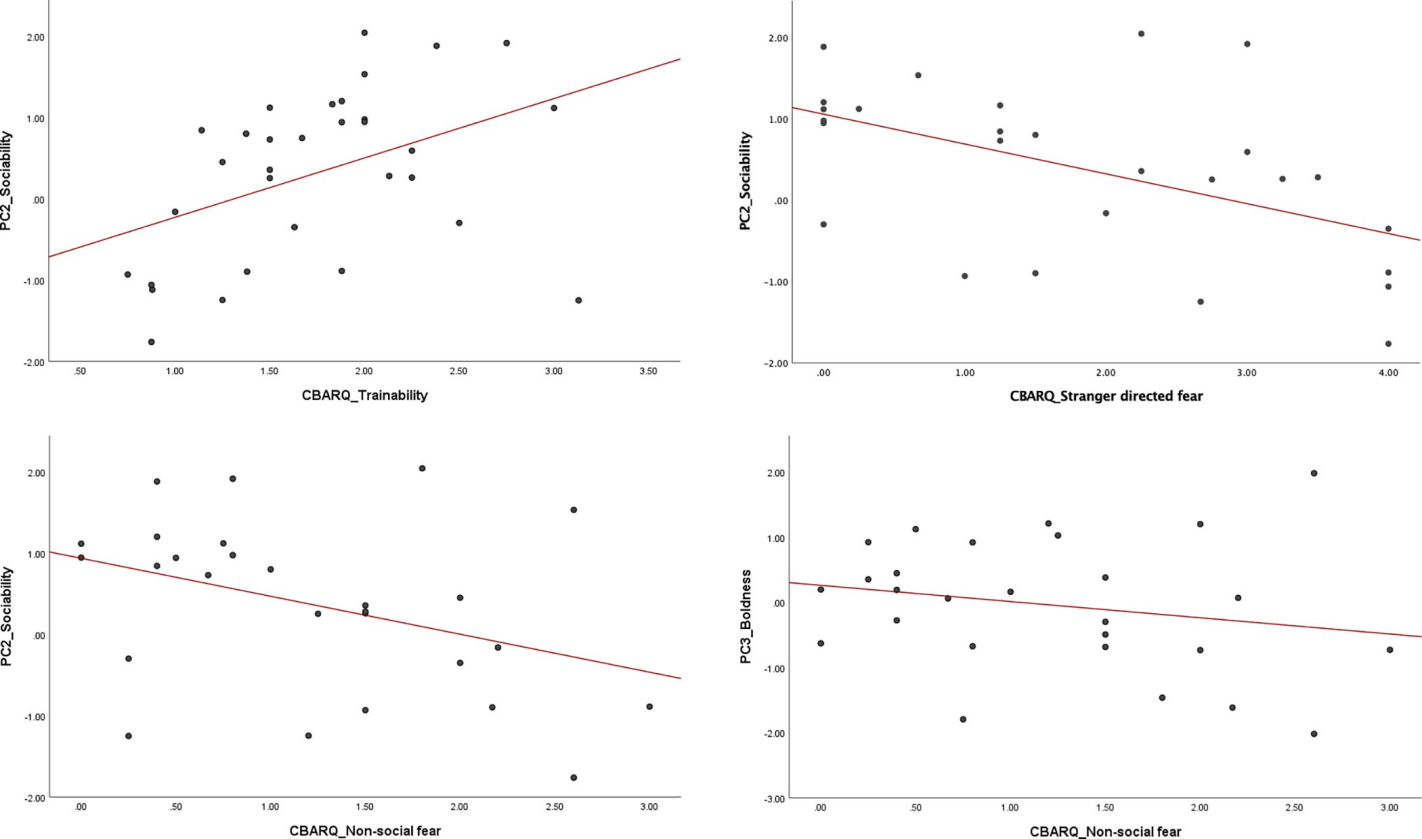
Relationship between pre- and post-adoption behavior. Scatterplot between PC2_sociability, extracted from the analysis of the in-kennel assessment variables, and the C-BARQ subscale trainability (a) stranger-directed fear (b) and non-social fear (c), and between PC3_boldness and CBARQ subscale non-social fear (d). Regression line represented in red.

Multiple regression analysis revealed a positive relationship between PC2_sociability and the CBARQ score trainability. Dogs with a higher score on sociability when assessed at the kennel were more likely to score higher on trainability at home (0.2577 ± 0.1093; p = 0.026; Fig 7A), whereas dogs with lower PC scores for sociability were more likely to show higher stranger-directed fear (-0.7678 ± 0.2454; p = 0.005; Fig 7B) and non-social fear scores (-0.4494 ± 0.1432; p = 0.005; Fig 7C) at home.

Similar results were reported for the scores on PC3_boldness and PC4_ responsiveness. Specifically, dogs scoring higher on PC3_boldness were less likely to have severe CBARQ scores for non-social fear (-0.3896 ± 0.1569; p = 0.021; Fig 7D), whereas dogs scoring higher on PC4_ responsiveness tended to be less likely to have severe CBARQ scores for stranger-directed fear (-0.4886 ± 0.2514; p = 0.065) and non-social fear (-0.2527 ± 0.1329; p = 0.071). No significant relationships were found between PC1_food interest and the CBARQ scores.

**Fig 8:**
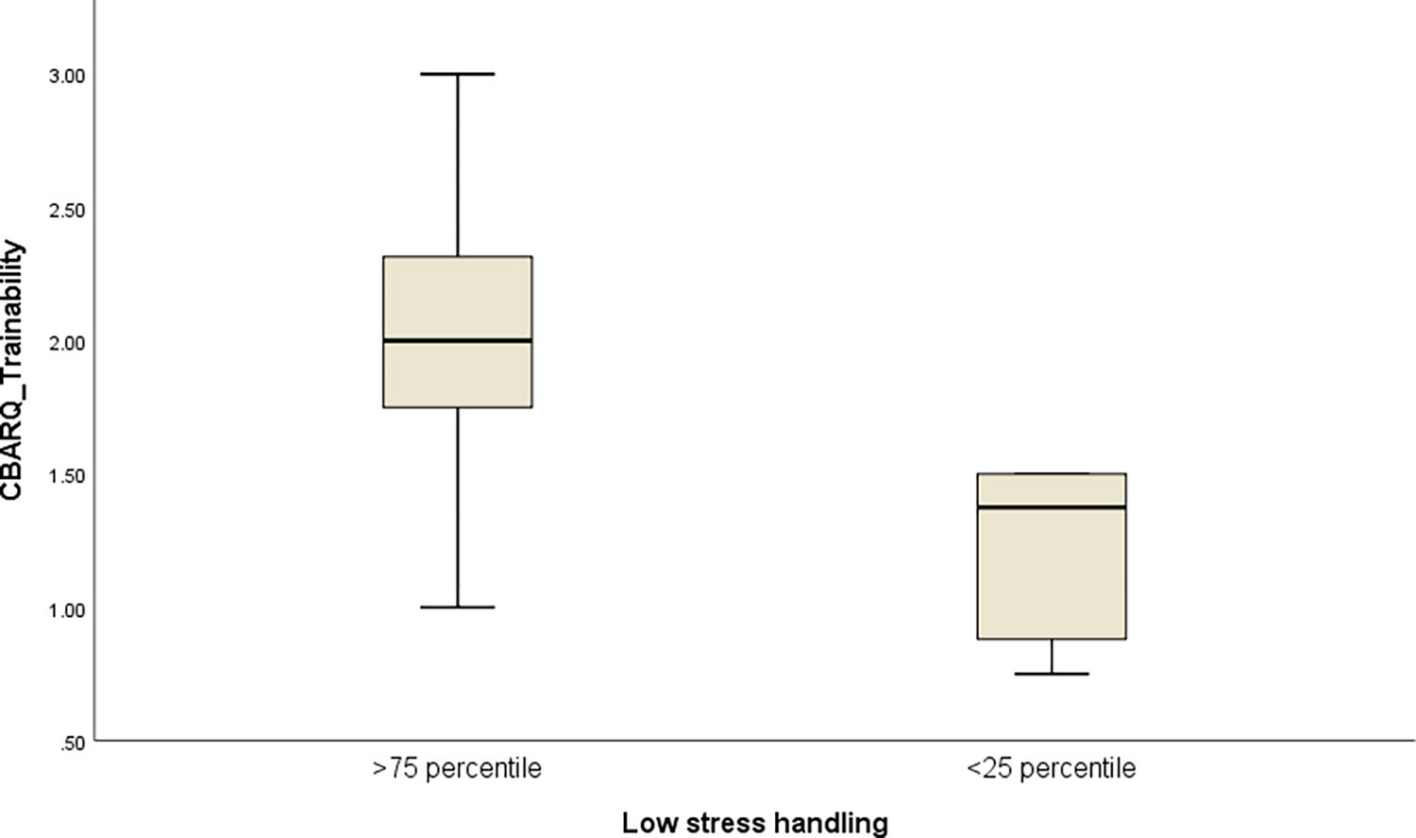
Relationship between management and post-adoption behavior. Boxplot representing the comparison for CBARQ trainability scores between dogs coming from kennels where the breeder was performing regular low-stress handling practices (>75th percentile) and dogs coming from kennels where this practice was rarely performed (<25th percentile). Values represented are: medians (bar within the box), upper and lower quartiles (borders of box), lowest and highest cases within 1.5 times the IQR (bottom and top whiskers).

When investigating if dogs’ CBARQ scores differed as a function of management practices in the kennels, we found that dogs received significantly higher CBARQ scores on trainability if the breeder used low-stress/consistent, gentle, and predictable handling regularly compared to those who used it rarely or never (U = 6.0, p = 0.014, Fig 8). No other significant differences were found for the other management components.

## Discussion

This study represents the first direct assessment of breeding dogs in both their CB kennels of origin as well as during their retirement and placement in adoptive homes. This is an important step toward understanding the relationship between the care and management practices experienced by dogs reared in such kennels and the implications for dog quality of life outside of the kennels, including their ability to transition from a kennel to a new home environment.

### Social and non-social fear in CB kennels and its implications for rehoming

The approach test showed that although over half of the dogs were non-fearful when approached by a tester from outside the pen (in line with previous similar studies, [10, 11]), as the test progressed and the interactions became more intense, the proportion of affiliative reactions (‘green’) decreased, and ambivalent reactions (‘yellow’) increased. It should be noted that in any setting, many, if not, most dogs would be uncomfortable with an unfamiliar person approaching and reaching for them without the presence of their owners or caretakers to reassure them. Nonetheless, these results may reflect the need for more and/or improved socialization and handling practices for dogs to be more comfortable with people approaching and attempting to interact with them given the need to tolerate such interactions to some degree in their kennels and in homes. For example, almost 90% of breeders reported that dogs were only exposed to unfamiliar visitors occasionally. In addition, most of the interactions with the dogs were limited to daily feeding and cleaning. Handling was associated with potentially stressful events such as grooming, health checks, and treatment administration, and only 34% of breeders reported spending time interacting with the dogs outside husbandry tasks. If dogs associated being reached for with negative experiences, it would make sense that increased aversion to the tester was observed when they opened the pen door and reached to touch the dogs.

A lower dog per caretaker ratio was associated with the likelihood of dogs scoring higher on sociability (Table 4). This confirms previous findings that a lower dog to caretaker ratio was associated with more affiliative scores during an approach test [2]. Having more staff (or fewer dogs) may allow for longer, higher quality time spent in each pen during husbandry chores, resulting in a better dog-caretaker relationship and in turn less fear. Interestingly, the fearful responses of the dogs toward an unfamiliar person during the in-kennel behavioral assessment also related to a higher likelihood of showing stranger-directed fear and non-social fear after rehoming (Fig 7B). Sociability has been previously found to be a consistent behavioral trait during short- and long-term test-retest reliability studies, making the assessment of dog fear levels toward people in kennels a potential predictor of post-adoption behavior [12, 19, 20]. These results highlight the important role gentle, positive handling and socialization practices at the kennel may play in dogs forming positive associations with people and generalizing these from familiar to unfamiliar people. Research underway by this group is further exploring how the type (i.e., quality) and quantity of specific behavioral management practices, such as socialization and handling, affect adult breeding dogs and whether this may generalize from caretakers to strangers.

The in-kennel behavioral assessment also allowed us to record the responses of the dogs towards non-social stimuli, including objects to which companion dogs in homes may commonly be exposed (e.g., dog toys, leash). Exposure to an inanimate object such as the plastic cone and a squeaky toy resulted in a close-to-chance distribution of responses (i.e., the dogs’ reactions equally distributed across the three scores). Most dogs reacted in a fearful manner to the opening of an umbrella (71.7%, Fig 2). This was expected as the umbrella test is typically performed to elicit a startle response. In this test, the umbrella was opened repeatedly up to 10 times to assess habituation (decreased or extinguished fear response). The dogs from our sample did not readily habituate to the opening of the umbrella, as the majority of subjects (64.2%) were still scored as fearful at the end of this test. Interestingly, the fake dog (commonly used as proxy measure for dog sociability) elicited very few fearful responses (7.7%) and mostly confident and friendly interactions (50%) or cautious interactions (42.3%), especially when compared to a non-social novel object as the plastic cone. This positive attitude toward a conspecific-like stimulus makes sense in light of the high level of exposure dogs have to others of their own kind in CB kennels. Often, dogs are turned out in exercise yards in large groups, and they are often group-housed. These results also align with previous studies where a fake dog was used in behavioral assessments and a higher occurrence of social behaviors was recorded compared to other inanimate objects not resembling a conspecific [13, 21, 22]. The responses to exposure to these different objects loaded together in a single behavioral category that was labelled boldness. Higher values on this component, corresponding to more exploratory and less fearful interactions with the stimuli, were found to be associated with lower non-social fear scores after adoption. The response to the umbrella test loaded on a different component labelled responsiveness. Similarly, higher scores on this component, corresponding to less fearful reactions towards startling stimuli, were associated with lower stranger-directed and non-social fear scores after rehoming. These findings suggest the usefulness of a pre-adoption assessment to identify individuals that may show more or fewer fear-related behaviors after adoption.

### Effect of dog demographic and management on behavior and health metrics in CB kennels

Demographic characteristics such as sex and breed, as well as management factors such as housing, origin, and facility were found to contribute to the variation in the dogs’ behavioral responses to the in-kennel assessment. For example, females were more likely to take treats throughout the test (higher PC1_food interest scores) than males. This may be due to a combination of higher energetic requirements of pregnancy and higher activity induced by group housing (compared to single housed males). Another hypothesis, however, is that taking treats from an unfamiliar person may also be a proxy measure of social fear and/or lack of exposure to novel food or being used to receiving treats directly from a person’s hand. Typically, in the CB kennels studied, males had less exposure to handling/caretaker interactions than females, while bitches received more interaction and handling around whelping, and more frequent monitoring happening during the peri-parturient period. Bitches were found to be more likely to react fearfully to a startling stimulus (lower PC4_responsiveness scores) than studs. This finding is consistent with most of the literature in which males are normally reported as bolder (i.e., less neophobic) than female dogs in different populations of working and pet dogs [23]. Collectively, these findings suggest the need to ensure equal attention to the handling and socialization of bitches and studs in CB kennels to ensure best outcomes in kennels and homes. Further research in these areas is needed.

Housing also appeared to affect the behavioral responses of dogs to our tests. For example, group-housed dogs scored lower (i.e., more fearfully) on PC4_responsiveness than pair- and singly-housed dogs, whereas singly-housed dogs scored higher on PC2_sociability than the other two groups. It is possible that group-housed dogs were more distressed when isolated from their group-mates, as was the case for dogs in our study during the behavioral assessment. Similarly, singly-housed dogs were more eager to interact socially when the opportunity was presented following isolation. In light of this, future studies should consider the effects of rehoming these dogs singly or to households without other dogs, as the absence of conspecifics may pose a challenge to them.

Age at entry to the kennel was related to fear responses observed. Dogs acquired as puppies at the kennel were more likely to explore and were less fearful when interacting with stimuli (i.e., scored higher on PC3_boldness) than dogs purchased as adults and dogs bred and raised at the kennel. This may be due to greater behavioral plasticity in puppies compared to adults. It should be noted that breed and facility also contributed to the explained variation of the behavioral components. Because our sample was not representative of all possible breeds and cross-breeds, nor all types of facility systems, these were included in the model as random factors.

The overall health of the dogs assessed in this study was good, supporting previous studies on dogs from US commercial breeding kennels [15, 24]. No increase in health problems was detected with age (Table 4). Dogs had normal body condition for the most part, and none were severely underweight, likely due to the common free-feeding system applied by all the breeders included in the study. The relatively small proportion (20%) of dogs scored as overweight did not show evidence of health concerns associated with this condition (e.g., reduced mobility, joint pain etc.). Dogs were mostly clean, with good coat condition and they were well groomed. Ocular discharge and tear staining appeared to be the most frequently recorded minor issues, with some breeds showing more tear staining than others (Fig 3) [25, 26].

One finding that was unexpected was the interaction between total health score and the dog:interaction variable. Specifically, when there were more dogs per caretaker, dogs were likely to be less healthy. Providing additional interaction, especially when the dog to caretaker ratio is lower, may offer an opportunity to monitor the individuals more closely and spot problems early. Finally, we found a significant effect of facility and breed on the total health score (S2 and S3 Tables in S1 File). The implementation of different management practices by breeders (some explored in this study, but other not explored, such as their preventative treatments and monitoring protocols) may have a direct effect on the prevalence of health problems recorded in a facility. Similarly, the effect of breed may indicate different susceptibility levels of some breeds compared to others. This should be explored in more depth by collecting information on dogs’ clinical histories and on the preventative treatments and veterinary care protocols applied at each kennel.

One month after adoption, the most common owner-reported health concerns were digestive problems (e.g., vomiting, diarrhea). Some owners also reported skin problems, external parasites (e.g., ear mites) and infections (especially ear infections). Although the total occurrence of each issue was low (≤ 5), this could still indicate an opportunity to refine veterinary preventative care and treatment protocols at some kennels. However, given the absence of a medical diagnosis, these issues could have developed after adoption, triggered for example by a change in diet or stress which is often cause of gastro-intestinal problems and allergies [27–30].

### Associations between management practices and rehoming outcomes

Data collected using the management questionnaire revealed that a portion of the breeders did not have protocols in place that would help prepare their dogs to transition to a family home. For example, over 50% of breeders did not leash train their dogs before rehoming. Additionally, exposure to unfamiliar visitors was only reported sporadically, and over 35% of breeders did not offer additional positive interactions outside of daily husbandry routines. These findings indicate where opportunities may exist for breeders to enhance their kennel management practices so as to facilitate positive rehoming outcomes for their retired breeding dogs.

An important factor in adoption success is the degree to which new owners’ expectations of the dogs they are considering adopting are realistic. Even though owners reported some behavioral and/or health issues, their satisfaction scores were overall high and no dogs were returned during this study. The latter finding was particularly interesting and bodes well for successful rehoming given that studies on dogs that were rehomed from shelters reported that most returns (>50%) occurred within the first month after adoption [5, 31]. Nonetheless, these findings should be cautiously interpreted as this was a convenience sample of owners who voluntarily agreed to participate in the study, and they may have been more prepared for and committed to the care of these dogs than owners in other studies. The owners in our study were also aware that they were adopting older dogs from commercial breeding facilities, and thus, they may have anticipated and planned to deal with health and behavior problems typical of senior dogs. Similar effects of awareness by adopters were observed by Marston et al. [32], who reported “compassion” or previous experience with a shelter dog as main reasons for adopting a dog from a shelter.

Overall, the severity of the most frequently reported behavioral problems with the CBARQ was low. In previous studies of dogs rehomed from kennels, house soiling was among the most frequently reported concerns by new adoptive owners [4, 5, 32]. Training difficulties were reported by 11 owners in this study (34.4%) and house-training challenges were reported by 18.8% (6) of respondents. The relatively small proportion of housebreaking issues may have been due to most dogs in this study having unrestricted indoor/outdoor access at the kennel, with opportunities to eliminate away from resting areas as is consistent with normal canine behavior [33]. However, some dogs may not generalize that behavior readily to a home environment with restricted outdoor access, and thus may need additional house-training. Previous findings have shown that dogs that were trained, including those that were leash trained and taught basic cues, such as ‘sit’ and ‘stay’, had higher chances of successful adoption [34]. The inconsistencies between the sampled kennels relative to having training protocols (Fig 4) may have correspondingly resulted in variation in success transitioning breeding dogs into homes where basic activities and interactions, including leash walking, are desirable and expected. However, lack of training at the kennels was not directly linked with lower trainability scores as reported by the new owners. This is not surprising as the trainability scale in the CBARQ is not defined by the dog’s knowledge of basic training, but by the dog’s predisposition to focus attention on a person and to interact and cooperate with that individual. Interestingly, the dog’s sociability score at the kennel was positively associated with the trainability score at home, and a similar relationship was found between receiving low stress/gentle handling by the breeders and a higher CBARQ trainability score. It therefore appears that having experienced positive and/or reward-based interactions at the kennel may have had an effect on perceived trainability of rehomed breeding dogs. Previous studies such as the one by Rooney and Cowan [35] have demonstrated that reward-based training methods increased the likelihood of future training success, such as the dog learning to perform a new task [35]. These findings suggest the importance of breeders promoting positive/gentle handling within the kennel. The finding that food interest was inversely related to the dog:interaction variable suggests that caretakers responsible for fewer dogs may have been able to spend more quality time with each individual, which included playing and giving treats. If so, dogs experiencing these types of interactions may have been less afraid, more motivated to interact with familiar and unfamiliar people, and more likely to accept treats from an unfamiliar person during testing. Training breeders on techniques such as gentle/low-stress handling [16], and advising them about the importance of regular positive interactions and training with their dogs outside of normal husbandry procedures may therefore contribute to the successful rehoming of retired breeding dogs.

When interpreting the results obtained from the rehomed dogs, a few limitations should be considered. First there was a small sample size due to the challenge of enrolling owners to participate in the follow-up study, which may reduce generalizability of the findings. Also, despite measures to reduce social desirability bias, it is not reasonable to believe that all such bias was entirely removed from the owner-based survey data and the short-term follow up that occurred one month later. We are currently conducting a study addressing most of these limitations, that includes in-home visits 12 months after adoption to permit direct physical and behavioral health assessment of the dogs as well as owner interviews.

In summary, dogs in CB kennels were found to be physically healthy. Most breeders provided basic enrichment and exercise to the adult dogs (such as chews, toys and regular access to exercise yard) and socialization to the puppies (including exposure to children, puppies from other litters, positive interaction and enrichment). Training and adult social interactions were less common. A moderate proportion of dogs showed fearful responses toward either social or non-social stimuli, confirming previous findings from this population of dogs [2, 11, 13]. Factors such as sex, social housing, and the number of dogs per caretaker ratio appeared to have an effect on the in-kennel behavior and health scores of the dogs. The latter factor was of particular interest. A lower dog to caretaker ratio was associated with better health scores and better sociability and food interest scores. It was difficult to determine whether the observed association was due to the lower number of dogs per caretaker affecting the quantity and/or quality of the interaction with individual dogs, or if breeders that were more knowledgeable about dogs’ welfare needs chose to have fewer dogs or more caretakers. Although our results showed some clear benefits of a lower dog to caretaker ratio, there are currently no published studies that have determined an optimal ratio. Such information is needed to inform breeder management practices and related standards of care for dogs in kennels.

## Conclusions

Higher levels of sociability (measured through PC2_sociability scores) in the kennels were associated with lower levels of social and non-social fear, and higher trainability after rehoming (measured through CBARQ subscales). This finding suggests that breeders may be able to improve dog welfare outcomes by attending closely not just to management of dogs but also to their genetic selection for sociability, as this may not just improve quality of life in the kennel but may also ease dogs’ post-retirement transition to homes. Even though this study did not specifically test the predictive validity of the in-kennel behavioral test, based on the current findings, a comprehensive behavioral assessment of rehoming candidates may help identify individuals at risk of experiencing difficulty transitioning to homes and inform early in-kennel interventions. These might include implementing more positive gentle contact with dogs, such as reward-based training and gentle handling, which appear to positively affect dogs’ willingness to engage with people. Improved understanding of breeders’ management practices and of the relationships between these and in-kennel welfare outcomes may offer many benefits to dogs in residence there. Further, they may help to reduce or avoid fear-related problems when dogs are retired and rehomed from CB kennels, and increase the likelihood of long-term rehoming success.

## Supporting information

S1 File Additional results. This document contains tables with additional results.
(DOCX)

S2 File Management questionnaire. The complete management questionnaire administered to the breeders is presented here.
(PDF)
