# Supplementary material for: Management and behavioral factors associated with rehoming outcomes of dogs retired from commercial breeding kennels
Source: PLoS One. 2023 Mar 2;18(3):e0282459. doi: 10.1371/journal.pone.0282459 (PMC9980791; doi:10.1371/journal.pone.0282459)
Supplement: S1 File — This document contains tables with additional results. (DOCX) [file pone.0282459.s001.docx]

**Supporting Information**

**Management and behavioral factors associated with rehoming outcomes of dogs retired from commercial breeding kennels**

Shanis Barnard^1*^, Hannah Flint^1^, Alessia Diana^1^, Traci Shreyer^1^, Aitor Arrazola^1^, James A. Serpell^2^, Candace Croney^1^

^1^Department of Comparative Pathobiology, Purdue University, 625 Harrison St., West Lafayette, IN, USA

^2^School of Veterinary Medicine, University of Pennsylvania, 3900 Delancey St., Philadelphia, PA, USA

^#a^Current Address: WALTHAM Petcare Science Institute, Freeby Lane, Waltham on the Wolds, Leicestershire, LE14 4RT

*Corresponding author: [barnard4@purdue.edu](mailto:barnard4@purdue.edu)

**Table 1S.** Number of subjects per breed and sex

| **#** | **Breed** | **Female** | **Male** | **Breed total** |
| --- | --- | --- | --- | --- |
| 1 | Alaskan Malamute | 3 | 2 | 5 |
| 2 | American Cocker Spaniel | 17 | 2 | 19 |
| 3 | American Eskimo Dog | 3 | 0 | 3 |
| 4 | Australian Shepherd | 13 | 4 | 17 |
| 5 | Bernese Mountain Dog | 2 | 1 | 3 |
| 6 | Bichapoo | 1 | 0 | 1 |
| 7 | Bichon Frise | 30 | 1 | 31 |
| 8 | Boston Terrier | 4 | 1 | 5 |
| 9 | Boxer | 5 | 0 | 5 |
| 10 | Bulldog | 6 | 0 | 6 |
| 11 | Bullmastiff | 14 | 3 | 17 |
| 12 | Cavalier King Charles Spaniel | 40 | 8 | 48 |
| 13 | Cocker Spaniel | 17 | 1 | 18 |
| 14 | Corgi | 3 | 1 | 4 |
| 15 | Coton de Tulear | 1 | 0 | 1 |
| 16 | Dachshund | 10 | 5 | 15 |
| 17 | French Bulldog | 18 | 6 | 24 |
| 18 | German Shepherd Dog | 1 | 0 | 1 |
| 19 | Golden Retriever | 21 | 4 | 25 |
| 20 | Goldendoodle | 5 | 0 | 5 |
| 21 | Great Dane | 11 | 5 | 16 |
| 22 | Havanese | 19 | 7 | 26 |
| 23 | Labrador Retriever | 25 | 5 | 30 |
| 24 | Lhasa Apso | 2 | 0 | 2 |
| 25 | Maltese | 10 | 5 | 15 |
| 26 | Miniature American Shepherd | 3 | 1 | 4 |
| 27 | Miniature Pinscher | 3 | 0 | 3 |
| 28 | Miniature Poodle | 6 | 5 | 11 |
| 29 | Miniature Schnauzer | 17 | 5 | 22 |
| 30 | Morkie | 1 | 0 | 1 |
| 31 | Neopolitan Mastiff | 13 | 2 | 15 |
| 32 | Newfoundland | 0 | 1 | 1 |
| 33 | Old English Sheepdog | 0 | 1 | 1 |
| 34 | Oripei | 1 | 0 | 1 |
| 35 | Pekingnese | 2 | 0 | 2 |
| 36 | Pomeranian | 13 | 2 | 15 |
| 37 | Pomsky | 0 | 1 | 1 |
| 38 | Pug | 4 | 0 | 4 |
| 39 | Rottweiler | 6 | 4 | 10 |
| 40 | Saint Bernard | 7 | 2 | 9 |
| 41 | Samoyed | 2 | 1 | 3 |
| 42 | Shetland Sheepdog | 13 | 1 | 14 |
| 43 | Shiba Inu | 5 | 1 | 6 |
| 44 | Shih Tzu | 35 | 9 | 44 |
| 45 | Siberian Husky | 22 | 6 | 28 |
| 46 | Silky terrier | 3 | 1 | 4 |
| 47 | Standard Poodle | 5 | 2 | 7 |
| 48 | Toy Poodle | 15 | 9 | 24 |
| 49 | Yorkshire Terrier | 13 | 5 | 18 |
|  | **Grand Total** | **470** | **120** | **590** |

**Figure 1S**

**Figure 1S.** **Dog housing.** Number of kennels housing male and female dogs singly, in pairs, or in groups (>3 dogs)

**Table 2S**. Estimates and SE of facilities for PC scores (i.e., Food motivation, Sociability, Boldness and Responsiveness) and total health

|  |  | **PC1 Food Motiv.** | | P value | **PC2 Sociability** | | P value | **PC3 Boldness** | | P value | **PC4 Responsiv.** | | P value | **Tot Health** | | P value |
| --- | --- | --- | --- | --- | --- | --- | --- | --- | --- | --- | --- | --- | --- | --- | --- | --- |
|  |  | Estimate | SE |  | Estimate | SE |  | Estimate | SE |  | Estimate | SE |  | Estimate | SE |  |
| **Facility** |  |  |  | = 0.0096 |  |  | = 0.0344 |  |  | = 0.0051 |  |  | = 0.2325 |  |  | = 0.0101 |
|  | Intercept | 0.1079 | 0.0461 |  | 0.0871 | 0.0479 |  | 0.1322 | 0.0514 |  | 0.0169 | 0.0231 |  | 0.1280 | 0.0551 |  |
|  | F1 | 0.3842^^^ | 0.1993 |  | 0.2998 | 0.1947 |  | -0.5141^*^ | 0.1977 |  | -0.0419 | 0.1151 |  | 0.1895 | 0.2316 |  |
|  | F2 | 0.1652 | 0.2182 |  | 0.1493 | 0.2251 |  | 0.1550 | 0.2019 |  | -0.0505 | 0.1171 |  | 0.0539 | 0.2775 |  |
|  | F3 | 0.0752 | 0.2181 |  | 0.0577 | 0.2326 |  | -0.2916 | 0.2084 |  | -0.0493 | 0.1176 |  | -0.0741 | 0.2765 |  |
|  | F4 | -0.3314 | 0.2050 |  | -0.0020 | 0.1976 |  | -0.5583^*^ | 0.2074 |  | -0.0726 | 0.1168 |  | 0.2082 | 0.2262 |  |
|  | F5 | -0.0757 | 0.2069 |  | 0.0508 | 0.1996 |  | 0.3000 | 0.1994 |  | 0.0132 | 0.1159 |  | 0.2940 | 0.2409 |  |
|  | F6 | 0.3481^^^ | 0.1970 |  | 0.4107^*^ | 0.1988 |  | 0.1558 | 0.1983 |  | -0.0253 | 0.1149 |  | -0.3304 | 0.2393 |  |
|  | F7 | -0.3096 | 0.1892 |  | -0.2859 | 0.1817 |  | 0.4578^*^ | 0.1957 |  | 0.0471 | 0.1139 |  | -0.3261 | 0.2063 |  |
|  | F8 | 0.4823^*^ | 0.1874 |  | -0.0171 | 0.1780 |  | -0.0689 | 0.1903 |  | -0.0007 | 0.1134 |  | -0.1864 | 0.2073 |  |
|  | F9 | 0.2203 | 0.2031 |  | -0.2743 | 0.1898 |  | -0.3403^^^ | 0.2001 |  | 0.0561 | 0.1160 |  | 0.2874 | 0.2266 |  |
|  | F10 | . | . |  | . | . |  | 0.3200 | 0.2202 |  | . | . |  | . | . |  |
|  | F11 | 0.0057 | 0.1989 |  | -0.3503^^^ | 0.1896 |  | 0.0509 | 0.2006 |  | 0.0231 | 0.1154 |  | -0.2746 | 0.2173 |  |
|  | F12 | 0.1223 | 0.2000 |  | 0.4287^*^ | 0.1915 |  | -0.1193 | 0.2042 |  | 0.0781 | 0.1156 |  | -0.1261 | 0.2227 |  |
|  | F13 | -0.6150^*^ | 0.2106 |  | 0.2513 | 0.2157 |  | 0.3022 | 0.1997 |  | 0.0501 | 0.1163 |  | 0.2274 | 0.2741 |  |
|  | F14 | 0.1842 | 0.1960 |  | -0.1006 | 0.1810 |  | -0.0770 | 0.1978 |  | -0.1247 | 0.1150 |  | -0.3757^^^ | 0.2111 |  |
|  | F15 | 0.0307 | 0.2029 |  | 0.0950 | 0.2026 |  | 0.1159 | 0.1947 |  | 0.0769 | 0.1150 |  | -0.1401 | 0.2457 |  |
|  | F16 | 0.3841^^^ | 0.2203 |  | 0.0374 | 0.2264 |  | 0.3825^^^ | 0.2127 |  | -0.0836 | 0.1175 |  | 0.0815 | 0.2780 |  |
|  | F17 | -0.0673 | 0.1996 |  | 0.1447 | 0.1934 |  | -0.4551^*^ | 0.1964 |  | 0.0383 | 0.1152 |  | -0.3161 | 0.2309 |  |
|  | F18 | -0.2889 | 0.2344 |  | 0.3058 | 0.2399 |  | 0.2791 | 0.2310 |  | 0.0570 | 0.1200 |  | -0.1662 | 0.3010 |  |
|  | F19 | -0.2021 | 0.2317 |  | -0.0492 | 0.2391 |  | -0.1795 | 0.2224 |  | 0.0439 | 0.1191 |  | 0.2890 | 0.3009 |  |
|  | F20 | 0.1981 | 0.1966 |  | -0.1349 | 0.1824 |  | -0.1374 | 0.2039 |  | -0.0589 | 0.1155 |  | -0.3711^^^ | 0.2120 |  |
|  | F21 | 0.0310 | 0.2419 |  | -0.1994 | 0.2048 |  | 0.2326 | 0.2117 |  | -0.0546 | 0.1202 |  | 0.0834 | 0.2675 |  |
|  | F22 | -0.2729 | 0.2086 |  | -0.0457 | 0.1827 |  | 0.5095^*^ | 0.2003 |  | 0.1055 | 0.1164 |  | 0.3171 | 0.2263 |  |
|  | F23 | 0.1150 | 0.2074 |  | -0.1749 | 0.1840 |  | -0.5608^*^ | 0.2068 |  | 0.0439 | 0.1166 |  | -0.1388 | 0.2246 |  |
|  | F24 | 0.1453 | 0.1944 |  | -0.0435 | 0.1834 |  | -0.0730 | 0.1933 |  | 0.0975 | 0.1143 |  | 0.2750 | 0.2149 |  |
|  | F25 | 0.1192 | 0.1920 |  | -0.3058^^^ | 0.1764 |  | -0.2175 | 0.1959 |  | -0.0109 | 0.1141 |  | -0.6130^*^ | 0.2074 |  |
|  | F26 | -0.1254 | 0.1972 |  | 0.2842 | 0.1865 |  | 0.1363 | 0.2021 |  | -0.0203 | 0.1155 |  | 0.0343 | 0.2195 |  |
|  | F27 | -0.4289^*^ | 0.2059 |  | -0.2279 | 0.1905 |  | -0.0899 | 0.2132 |  | -0.0394 | 0.1174 |  | 0.2300 | 0.2199 |  |
|  | F28 | -0.1438 | 0.1939 |  | 0.00151 | 0.1819 |  | 0.3155 | 0.2014 |  | -0.0626 | 0.1152 |  | 0.2205 | 0.2065 |  |
|  | F29 | -0.0841 | 0.1896 |  | -0.3134^^^ | 0.1739 |  | 0.1780 | 0.1942 |  | -0.0410 | 0.1138 |  | 0.3683^^^ | 0.2058 |  |
|  | F30 | -0.0659 | 0.2263 |  | 0.0081 | 0.2438 |  | -0.2082 | 0.1984 |  | 0.0054 | 0.1178 |  | 0.2792 | 0.2520 |  |

Statistically different from the intercept = ^*^P < 0.05; ^^^0.10 < P < 0.05

**Table 3S**. Estimates and SE of breeds for PC scores (i.e., Food motivation, Sociability, Boldness and Responsiveness) and total health

|  |  | **PC1 Food Motiv.** | | P value | **PC2 Sociability** | | P value | **PC3 Boldness** | | P value | **PC4 Responsiv.** | | P value | **Tot_Health** | | P value |
| --- | --- | --- | --- | --- | --- | --- | --- | --- | --- | --- | --- | --- | --- | --- | --- | --- |
|  |  | Estimate | SE |  | Estimate | SE |  | Estimate | SE |  | Estimate | SE |  | Estimate | SE |  |
| **Breed** |  |  |  | = 0.1294 |  |  | = 0.0276 |  |  | = 0.0650 |  |  | = 0.3429 |  |  | = 0.0048 |
|  | Intercept | 0.0268 | 0.0237 |  | 0.0903 | 0.0471 |  | 0.0414 | 0.0274 |  | 0.0101 | 0.0250 |  | 0.2114 | 0.0816 |  |
|  | Alaskan Malamute | 0.0141 | 0.1541 |  | 0.0215 | 0.2471 |  | -0.0966 | 0.1852 |  | 0.0038 | 0.0983 |  | -0.1793 | 0.3354 |  |
|  | American Cocker Spaniel | -0.1488 | 0.1348 |  | -0.1074 | 0.1823 |  | -0.0651 | 0.1549 |  | -0.0215 | 0.0929 |  | 0.0192 | 0.2247 |  |
|  | Australian Shepherd | -0.0403 | 0.1416 |  | 0.1631 | 0.1998 |  | -0.1264 | 0.1564 |  | -0.0204 | 0.0939 |  | -0.1409 | 0.2560 |  |
|  | Bichon Frise | -0.2000 | 0.1283 |  | -0.2596 | 0.1704 |  | 0.0232 | 0.1386 |  | -0.0382 | 0.0902 |  | 0.4390^*^ | 0.2088 |  |
|  | Boston Terrier | 0.0314 | 0.1535 |  | 0.1074 | 0.2437 |  | 0.0962 | 0.1846 |  | 0.0354 | 0.0982 |  | 0.1065 | 0.3284 |  |
|  | Boxer | -0.0918 | 0.1547 |  | 0.1595 | 0.2490 |  | 0.1055 | 0.1845 |  | 0.0374 | 0.0986 |  | -0.1371 | 0.3371 |  |
|  | Bulldog | -0.0078 | 0.1531 |  | 0.1361 | 0.2436 |  | 0.0327 | 0.1820 |  | 0.0302 | 0.0979 |  | 0.3842 | 0.3312 |  |
|  | Bullmastiff | -0.0502 | 0.1532 |  | -0.0510 | 0.2411 |  | -0.1559 | 0.1639 |  | 0.0263 | 0.0956 |  | 0.4772 | 0.3306 |  |
|  | C.K. Charles Spaniel | 0.1605 | 0.1175 |  | 0.2671^^^ | 0.1458 |  | 0.0801 | 0.1237 |  | 0.0379 | 0.0853 |  | 0.6572^*^ | 0.1805 |  |
|  | Cocker Spaniel | 0.0827 | 0.1387 |  | -0.1639 | 0.1924 |  | -0.0962 | 0.1549 |  | 0.0128 | 0.0933 |  | 0.7836^*^ | 0.2431 |  |
|  | Dachshund | 0.0598 | 0.1421 |  | -0.2491 | 0.2000 |  | 0.1204 | 0.1605 |  | -0.0384 | 0.0948 |  | -0.6688^*^ | 0.2575 |  |
|  | French Bulldog | 0.1053 | 0.1474 |  | 0.0364 | 0.2187 |  | 0.2550^^^ | 0.1546 |  | -0.0524 | 0.0939 |  | -0.1495 | 0.2875 |  |
|  | Golden Retriever | -0.0689 | 0.1309 |  | 0.3797^*^ | 0.1726 |  | -0.3255^*^ | 0.1444 |  | -0.0526 | 0.0909 |  | 0.0669 | 0.2140 |  |
|  | Goldendoodle | 0.0483 | 0.1522 |  | -0.1173 | 0.2380 |  | 0.0276 | 0.1846 |  | 0.0567 | 0.0981 |  | -0.0431 | 0.3158 |  |
|  | Great Dane | -0.1165 | 0.1501 |  | 0.0933 | 0.2326 |  | 0.1455 | 0.1585 |  | -0.0012 | 0.0966 |  | 0.2087 | 0.3167 |  |
|  | Havanese | -0.0772 | 0.1282 |  | -0.0499 | 0.1667 |  | 0.0040 | 0.1434 |  | -0.0622 | 0.0905 |  | 0.3325^^^ | 0.2066 |  |
|  | Labrador Retriever | 0.0788 | 0.1382 |  | 0.1160 | 0.1899 |  | 0.1097 | 0.1408 |  | 0.0251 | 0.0914 |  | -0.2916 | 0.2463 |  |
|  | Maltese | 0.0448 | 0.1382 |  | -0.2228 | 0.1911 |  | -0.0396 | 0.1600 |  | 0.0644 | 0.0940 |  | 0.0279 | 0.2405 |  |
|  | Miniature Poodle | 0.0870 | 0.1442 |  | -0.2851 | 0.2086 |  | -0.0892 | 0.1706 |  | -0.0052 | 0.0959 |  | 0.1096 | 0.2617 |  |
|  | Miniature Schnauzer | 0.0457 | 0.1306 |  | -0.4062^*^ | 0.1721 |  | 0.0449 | 0.1493 |  | -0.0109 | 0.0917 |  | -0.2766 | 0.2151 |  |
|  | Neopolitan Mastiff | -0.0718 | 0.1535 |  | 0.3170 | 0.2419 |  | 0.1159 | 0.1677 |  | 0.0341 | 0.0960 |  | -0.2745 | 0.3307 |  |
|  | Pomeranian | -0.0047 | 0.1426 |  | 0.2801 | 0.2041 |  | -0.2053 | 0.1615 |  | 0.0369 | 0.0949 |  | -0.0231 | 0.2572 |  |
|  | Rottweiler | 0.0549 | 0.1486 |  | 0.3442 | 0.2246 |  | 0.1322 | 0.1733 |  | -0.0441 | 0.0966 |  | -0.1479 | 0.2841 |  |
|  | Saint Bernard | -0.0364 | 0.1504 |  | 0.1672 | 0.2340 |  | 0.1518 | 0.1735 |  | 0.0312 | 0.0968 |  | 0.1668 | 0.3185 |  |
|  | Shetland Sheepdog | 0.0391 | 0.1511 |  | 0.0142 | 0.2367 |  | -0.0808 | 0.1631 |  | -0.0341 | 0.0955 |  | -0.5035^^^ | 0.3115 |  |
|  | Shiba Inu | 0.0755 | 0.1521 |  | 0.2367 | 0.2375 |  | -0.0230 | 0.1831 |  | 0.0349 | 0.0979 |  | -0.7551^*^ | 0.3160 |  |
|  | Shih Tzu | -0.1137 | 0.1141 |  | -0.1710 | 0.1403 |  | 0.0142 | 0.1255 |  | 0.0164 | 0.0854 |  | 0.3952^*^ | 0.1725 |  |
|  | Siberian Husky | 0.0542 | 0.1465 |  | -0.0173 | 0.2142 |  | 0.0322 | 0.1435 |  | -0.0229 | 0.0935 |  | -0.2801 | 0.2818 |  |
|  | Standard Poodle | 0.0277 | 0.1510 |  | -0.0862 | 0.2329 |  | -0.1223 | 0.1790 |  | -0.0255 | 0.0974 |  | -0.5532^^^ | 0.3076 |  |
|  | Toy Poodle | 0.0256 | 0.1281 |  | -0.3700^*^ | 0.1662 |  | -0.1664 | 0.1465 |  | -0.0364 | 0.0910 |  | 0.4428^*^ | 0.2051 |  |
|  | Yorkshire Terrier | -0.0073 | 0.1353 |  | -0.2825 | 0.1834 |  | 0.1012 | 0.1542 |  | -0.0175 | 0.0929 |  | -0.1929 | 0.2299 |  |

Statistically different from the intercept = ^*^P < 0.05; ^^^0.10 < P < 0.05

**Figure 2S**


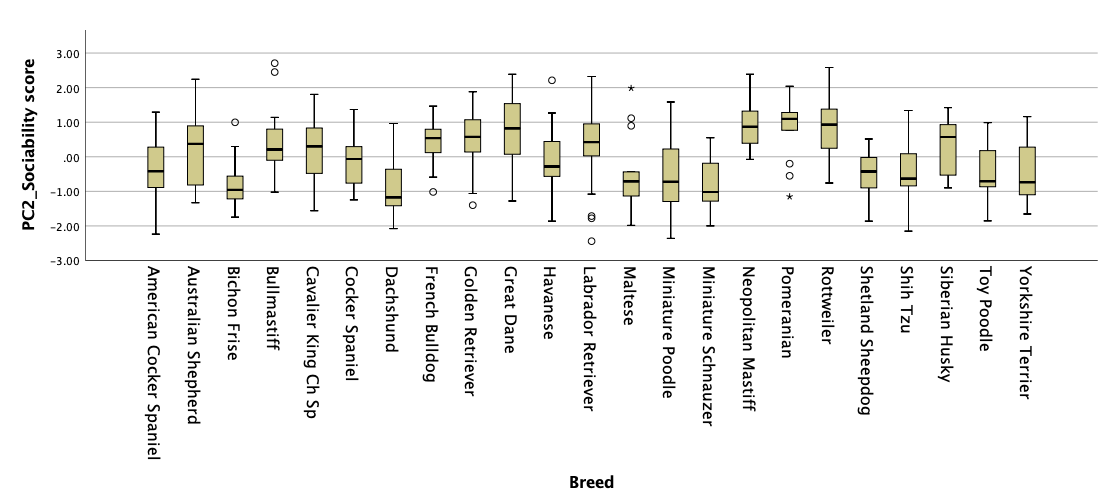


**Figure 2S.** **Behavioral variation by breed.** Boxplots illustrating between and within breed variation for PC2_Sociability scores extracted by the PCA. Only breeds with 10 or more individuals are represented.
