# Supplementary material for: Management and behavioral factors associated with rehoming outcomes of dogs retired from commercial breeding kennels
Source: PLoS One. 2023 Mar 2;18(3):e0282459. doi: 10.1371/journal.pone.0282459 (PMC9980791; doi:10.1371/journal.pone.0282459)
Supplement: S2 File — The complete management questionnaire administered to the breeders is presented here. (PDF) [file pone.0282459.s002.pdf]

BREEDER QUESTIONNAIRE

Please note all the information provided and all personal identifying information will be kept strictly confidential.

Any information collected will be used for scientific purposes and may be shared in an anonymous form.

Please note that there is no right or wrong answer. The questionnaire merely aims to record the management practices performed on this premise. The information provided will be essential for the purpose of the study.

FACILITY INFORMATION:

a) Kennel ID # ☐

b) Number of adult dogs at the facility ☐

c) Breeds:

☐

d) Are **female** dogs: single ☐ pair ☐ or group ☐ housed

If group housed how many/pen (average)? ☐

Are **male** dogs: single ☐ pair ☐ or group ☐ housed

If group housed how many/pen (average)? ☐

e) How many caretakers provide **daily routine** care to the dogs?

Adults (18+) ☐ Young (12-18) ☐ Children (under 12) ☐

f) How many caretakers interact with the dogs daily, **outside routine care** (e.g. for playing, socializing, training)?

Adults (18+) ☐ Young (12-18) ☐ Children (under 12) ☐

1 Management practices overview

[*Never/rarely* = happens inconsistently; *Sometimes* = every other week or a few times per month; *Often* = once or twice per week; or *Always* = daily (5-7 days per week)]

a) Are dogs given some form of **enrichment** (e.g. chews, toys, social interaction)? Y / N

○ Never/Rarely ○ Sometimes ○ Often ○ Always ○ I don't know

b) Are **adult** dogs provided some form of **socialization** (e.g. exposure to visitors, new places/objects)? Y / N

○ Never/Rarely ○ Sometimes ○ Often ○ Always ○ I don't know

c) Are **puppies** provided some form of **socialization** (e.g. exposure to visitors, new places/objects)? Y / N

○ Never/Rarely ○ Sometimes ○ Often ○ Always ○ I don't know

d) Does the kennel implement **early puppy handling** protocols (e.g. bio-sensor)? Y / N

○ Never/Rarely ○ Sometimes ○ Often ○ Always ○ I don't know

e) Are dogs provided **positive caretaker interaction** beyond husbandry (e.g. play sessions, training, petting)? Y / N

○ Never/Rarely ○ Sometimes ○ Often ○ Always ○ I don't know

f) Are dogs provided **exercise** and play opportunities (e.g. exercise outside home pen, training/sport activities)? Y / N

○ Never/Rarely ○ Sometimes ○ Often ○ Always ○ I don't know

g) Do dogs receive **training** (e.g. basic commands, walk on leash)? Y / N

○ Never/Rarely ○ Sometimes ○ Often ○ Always ○ I don't know

h) Are dogs **rehomed** at the end of their breeding careers? Y / N

○ Never/Rarely ○ Sometimes ○ Often ○ Always ○ I don't know

i) Does the **kennel design** incorporate features that encourage expression of dogs' natural behavior (e.g. built in ramps, stairs, denning areas)?

○ Yes ○ No ○ I don't know

List features:

2 Management practices details

a) Which of the following **enrichments** is normally provided to your dogs? (check all that apply):

○ N/A

**Enrichment**	**Examples**	**Yes**	*Is it alternated?* **D**aily, **W**eekly, **O**ccasionally, **N/A** *(circle)*
Nutritional	Puzzle Feeder (e.g. Kong with peanut butter)		D W O N/A
	Chew (e.g. hoof, bone)		D W O N/A
Physical environment	Toys (e.g. ball)		D W O N/A
	Surfaces (e.g. artificial and natural)		
	Complex kennel design (e.g. built in ramps, stairs, denning areas)		
	Scents for the dogs to sniff (e.g. lavender, chamomile, rabbit)		D W O N/A
Other (describe)	e.g. visitors, novel objects, radio, tv		

b) Which of the following **socialization** applications are used for **ADULT** dogs? (check all that apply):

○ N/A

Type	Daily	Weekly	Occasionally	Describe
Exposure to unfamiliar visitors				
Interactions w caretakers outside routine (e.g. play, pet, handing treats)				
Exposure to new places (e.g. yard, house, office)				
Exposure to new objects (e.g. umbrellas, furniture, bags)				
Exposure to different sounds (e.g. music, thunderstorms, vacuums)				
Other (describe)				

c) Which of the following **socialization and early handling** interventions are used for **PUPPIES**? (check all that apply):

○ N/A

Type	Daily	Weekly	Occasionally	Describe
Early (0-21 days of life) handling/stimulation (stroke, tickle, hold)				
Exposure to crating				
Exposure to unfamiliar visitors				
Interactions with caretakers outside routine (e.g. play, pet, handing treats)				
Exposure to new places (e.g. yard, house, office, puppy pen)				
Exposure to new objects (e.g. umbrellas, furniture, bags)				
Exposure to other puppies (not from litter)				
Exposure to sounds (e.g. music, banging metal)				

Other (describe e.g. flooring)				

a) Which of the following is performed when **handling/moving** the dogs? (check all that apply):

Procedure	Always	Sometimes	Never	NOTES
Feeding treats during handling/exams				
Fully supporting body weight when carrying the dog				
Speaking gently in a calm tone to the dogs				
Handling the dogs inside their home pen				
Handling dogs in a separate quiet exam area (avoiding raised voices, excess noise)				
Stroking/petting dogs during handling				
Using pain relief when painful procedures are performed (e.g. declaw removal, tail docking, dental extractions, surgeries)				
Using sedatives/anxiety reducing medications or techniques (e.g. pheromones, essential oils, aromatherapy)				

b) If dogs must be caught for **handling/moving,** the following approaches may be used (select all that apply):

Procedure	Always	Sometimes	Never	NOTES
Reaching for their collars				
Reaching for their coat, neck, legs or other body parts				
Calling them				
Offering a treat				
Other (describe)				

c) How are dogs **moved** from one area to another? (check all that apply):

Procedure	Always	Sometimes	Never	NOTES
On leash				
Carrying by fully supporting them				
Carrying by the scruff				
Dragging, sliding them across the floor				
Other (describe)				

d) Which of the following **exercise** activities are provided to the dogs? (check all that apply):

○ N/A

Exercise	Daily	Weekly	Occasionally	NOTES
Free access to outdoor run (part of home pen)				
Limited access to outdoor run (part of home pen)				
Access to outdoor exercise yard				
Group play with dogs other than pen-mates				
Play with people (e.g. fetch)				
Sport (e.g. agility)				
Leash walk				
Other (describe)				

e) If **training** is done, which of the following occur? (check all that apply):

○ N/A

Training	Daily	Weekly	Occasionally	Describe
Leash walking				
Basic commands (e.g. sit)				

Reward quiet behavior (give treat when dog is on all fours, relaxed, not barking etc.)				
Crate training/alone-time				
Training using bark control device (describe type)				
Other training practice (e.g. house breaking)				

NOTES:
